# Unifying Viral Genetics and Human Transportation Data to Predict the Global Transmission Dynamics of Human Influenza H3N2

**DOI:** 10.1371/journal.ppat.1003932

**Published:** 2014-02-20

**Authors:** Philippe Lemey, Andrew Rambaut, Trevor Bedford, Nuno Faria, Filip Bielejec, Guy Baele, Colin A. Russell, Derek J. Smith, Oliver G. Pybus, Dirk Brockmann, Marc A. Suchard

**Affiliations:** 1 Department of Microbiology and Immunology, KU Leuven, Leuven, Belgium; 2 Institute of Evolutionary Biology, University of Edinburgh, Edinburgh, United Kingdom; 3 Fogarty International Center, National Institutes of Health, Bethesda, Maryland, United States of America; 4 Department of Zoology, University of Cambridge, Cambridge, United Kingdom; 5 World Health Organization Collaborating Center for Modeling, Evolution, and Control of Emerging Infectious Diseases, Cambridge, United Kingdom; 6 Department of Virology, Erasmus Medical Centre, Rotterdam, Netherlands; 7 Department of Zoology, University of Oxford, Oxford, United Kingdom; 8 Engineering Sciences and Applied Mathematics, Northwestern University, Evanston, Illinois, United States of America; 9 Northwestern Institute on Complex Systems, Evanston, Illinois, United States of America; 10 Robert-Koch-Institute, Berlin, Germany; 11 Departments of Biomathematics and Human Genetics, David Geffen School of Medicine, University of California, Los Angeles, California, United States of America; 12 Department of Biostatistics, UCLA Fielding School of Public Health, University of California, Los Angeles, California, United States of America; Imperial College London, United Kingdom

## Abstract

Information on global human movement patterns is central to spatial epidemiological models used to predict the behavior of influenza and other infectious diseases. Yet it remains difficult to test which modes of dispersal drive pathogen spread at various geographic scales using standard epidemiological data alone. Evolutionary analyses of pathogen genome sequences increasingly provide insights into the spatial dynamics of influenza viruses, but to date they have largely neglected the wealth of information on human mobility, mainly because no statistical framework exists within which viral gene sequences and empirical data on host movement can be combined. Here, we address this problem by applying a phylogeographic approach to elucidate the global spread of human influenza subtype H3N2 and assess its ability to predict the spatial spread of human influenza A viruses worldwide. Using a framework that estimates the migration history of human influenza while simultaneously testing and quantifying a range of potential predictive variables of spatial spread, we show that the global dynamics of influenza H3N2 are driven by air passenger flows, whereas at more local scales spread is also determined by processes that correlate with geographic distance. Our analyses further confirm a central role for mainland China and Southeast Asia in maintaining a source population for global influenza diversity. By comparing model output with the known pandemic expansion of H1N1 during 2009, we demonstrate that predictions of influenza spatial spread are most accurate when data on human mobility and viral evolution are integrated. In conclusion, the global dynamics of influenza viruses are best explained by combining human mobility data with the spatial information inherent in sampled viral genomes. The integrated approach introduced here offers great potential for epidemiological surveillance through phylogeographic reconstructions and for improving predictive models of disease control.

## Author Summary

What explains the geographic dispersal of emerging pathogens? Reconstructions of evolutionary history from pathogen gene sequences offer qualitative descriptions of spatial spread, but current approaches are poorly equipped to formally test and quantify the contribution of different potential explanatory factors, such as human mobility and demography. Here, we use a novel phylogeographic method to evaluate multiple potential predictors of viral spread in human influenza dynamics. We identify air travel as the predominant driver of global influenza migration, whilst also revealing the contribution of other mobility processes at more local scales. We demonstrate the power of our inter-disciplinary approach by using it to predict the global pandemic expansion of H1N1 influenza in 2009. Our study highlights the importance of integrating evolutionary and ecological information when studying the dynamics of infectious disease.

## Introduction

The emergence and worldwide dispersal of novel human pathogens is increasingly challenging global public health [1]. Notable recent examples include novel influenza strains, severe acute respiratory syndrome (SARS) virus and Methicillin-resistant *Staphylococcus aureus*, which all exploit today's complex and voluminous transport networks to rapidly disseminate in a globalized world. In the context of human infectious diseases, the worldwide air transportation network is by far the best studied system of global mobility [2]. Air travel likely drives the global circulation of seasonal influenza A (H3N2) viruses [3], and may explain seasonal dynamics in the absence of locally-persistent strains between epidemic seasons. Retrospective modeling of the ‘Hong Kong flu’ H3N2 pandemic in 1968 indicates that the virus spread through a global network of cities interconnected by air travel [4]. Numerous modeling and simulation studies have subsequently explored the potential influence of air travel on influenza virus spread, e.g. [5]–[8], but few have attempted to verify such models against underlying empirical data on human movement patterns [9].

Two studies on the timing and rate of seasonal influenza transmission across the United States of America (USA) highlight the difficulty of using standard epidemiological data to disentangle the relative contributions of different human transportation systems to influenza spread. Using weekly time series of excess mortality due to pneumonia and influenza (P&I), Viboud et al. [9] demonstrated that the patterns of timing and incidence of outbreaks across the continental USA are most strongly associated with rates of movement of people to and from their workplaces, and to a lesser extent with the distance between locations and various measures of domestic transportation. In contrast, Brownstein et al. [10] concluded that the rate of inter-regional spread and timing of influenza in the USA, as measured using weekly P&I mortality statistics, is predicted by domestic airline travel volume in November. These discordant findings generated significant debate [11], especially in the context of a potential pandemic of pathogenic influenza [12], which would require rapid decisions to be made on the implementation of travel restrictions.

As a historical record of epidemic spread, viral genetic sequence data may offer a valuable source of information for the empirical verification of epidemiological models. Several studies have demonstrated their utility and power, for example by revealing the genetic dynamics of influenza A H3N2 seasonality [13] and the spatial patterns of global H3N2 circulation [3], [14]. More generally, it is recognized that the genetic diversity of rapidly evolving viruses like influenza should be analysed in a framework that unifies evolutionary and ecological dynamics [15]. Current attempts to reconstruct viral spread through time and space from genetic data, however, typically fit parameter-rich models to sparse spatial data and result in phylogeographic patterns that are difficult to relate directly to underlying ecological processes [16]. Together with potential sampling bias, this complicates phylogeographic tasks, such as the characterization of source-sink dynamics in seasonal influenza. It is therefore unsurprising that different studies on the global circulation of H3N2 are sometimes inconsistent [3], [14], [17], despite the importance of such work for influenza surveillance and vaccine strain selection.

Here we use a model-based approach to explicitly tests spatial epidemiological hypotheses by integrating empirical data on human movement patterns with viral genetic data. This framework enables us to measure the relative contribution of different predictive variables to viral spatial spread. We apply this approach to seasonal H3N2 dynamics and use it to identify key drivers of the global dissemination of influenza viruses. Analysis of different sampling schemes, including one that represents the community structure in global air transportation, provides consistent support for air travel governing the spatial dynamics of seasonal H3N2 infections. Using epidemiological simulations, we further demonstrate that estimates resulting from the merger of human air travel and H3N2 influenza genetics best capture the observed global expansion of pandemic H1N1 influenza in 2009.

## Methods

### Sequence data

We complemented a previously collected hemagglutinin sequence data set, comprising 1,441 sequences sampled globally from 2002 to 2007 [3], with publicly available sequences sampled within the same time interval. The allocation of the sequence data into 15 and 26 geographic regions as well as into 14 air communities is described in detail in Supporting information Text S1.

### Air transportation data and modularity maximization

The worldwide air transportation network is defined by a passenger flux matrix that quantifies the number of passengers traveling between each pair of airports. We use a dataset provided by OAG (Official Airline Guide) Ltd. (http://www.oag.com), containing 4,092 airports and the number of seats on scheduled commercial flights between pairs of airports during the years 2004–2006. We take the number of seats on scheduled commercial flights from airport *i* to *j* to be proportional to the number of passengers traveling.

To identify air transportation communities, we approximate a maximal-modularity subdivision of the 1,227-largest-airport network by employing a recently described stochastic Monte-Carlo approach [18]. Modularity provides a measure of how well the connectivity of a network is described by partitioning its nodes into non-overlapping groups; for a definition we refer to [19]. For any given partition, modularity will be high if connectivity within groups is high and connectivity among groups is low. For large networks, a variety of methods have been introduced to approximate their optimal subdivision. The method we employ here generates an ensemble of high modularity subdivisions and computes the consensus in this ensemble by superposition. For further details we refer to [18], [20] and in Text S1 we describe how we incorporate subdivision uncertainty in our phylogeographic approach.

### Phylogeographic inference and hypothesis testing

We employ a novel approach to simultaneously reconstruct spatiotemporal history and test the contribution of potential predictors of spatial spread. The approach extends a recently developed Bayesian method of phylogeographic inference [21] into a generalized linear model (GLM), by parameterizing each rate of among-location movement in the phylogeographic model as a log linear function of various potential predictors. For each predictor *j*, the GLM parameterization includes a coefficient 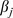, which quantifies the contribution or effect size of the predictor (in log space), and a binary indicator variable 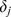, that allows the predictor to be included or excluded from the model. We estimate the 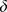 variables using a Bayesian stochastic search variable selection (BSSVS) [22], [23], resulting in an estimate of the posterior inclusion probability or support for each predictor. This approach uses the data to select the explanatory variables and their effect sizes from a pre-defined set of predictors that can explain the phylogenetic history of among-location movement while simultaneously reconstructing the ancestral locations in the evolutionary history. In Text S1, we (i) provide more mathematical detail of the GLM model, (ii) describe novel transition kernels for efficient statistical inference, (iii) propose prior specifications and (iv) explain how Bayes factors can be calculated for each predictor based on 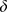 estimates. The method introduced here is implemented in the BEAST software package [24].

The GLM approach offers many statistical advantages over other approaches [25] in efficiently testing spatial hypotheses (see Text S1 for a detailed comparative analysis). Commonly-used Bayesian measures of model fit (such as marginal likelihood estimation using the harmonic mean), which can be applied to models with among-location movement rates fixed to a particular predictor, have been shown to perform poorly [26]–[28]. Although more accurate alternatives have recently been proposed [26]–[28], they are computationally prohibitive on large data sets such as those studied here. Importantly, the previous approach provides only a relative ranking of different models and, unlike the GLM model, cannot identify which of the top-ranked predictors need to be jointly considered as explanatory variables. A further advantage of the GLM approach is that in addition to providing a measure of support for each predictor, it can also quantify the contribution or effect size of each predictor by estimating the associated coefficients (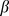).

For the spread of seasonal influenza, we consider several potential predictors of global migration, including different log-transformed measures of geographical distance, absolute latitude, air transportation data, demographic and economic data, viral surveillance data, antigenic evolution and sequence sample sizes (described in more detail in Text S1). Text S1 also reports the evolutionary and demographic models used in BEAST and describes how phylogenetic uncertainty is approximated during phylogeographic inference.

Phylogeographic movement events among locations are modeled by a continuous-time Markov chain (CTMC) process along each branch of the viral phylogeny. Although both the transitions among locations (Markov jumps) and the waiting times between transitions (Markov rewards) are not directly observed, posterior expectations of these values can be efficiently computed [29], [30]. Here, we implement posterior inference of the complete Markov jump history through time in BEAST and use these estimates to assess the source-sink dynamics of influenza and to evaluate the predictive performance of phylogeographic models.

### Comparing migration rate models using epidemiological simulations

To compare the performance of different migration rate models in predicting global pandemic spread, we simulate a stochastic meta-population susceptible-infected-recovered (SIR) model with *n* = 14 populations, matching the 14 air communities analyzed in the phylogeographic model. The model tracks the number of susceptible (*S*), infected (*I*) and recovered (*R*) individuals in each population each day of the simulation. The simulations begin with a single initial infection in Mexico on January 5th 2009 [31]. Infection spreads through mass-action within each population according to the following epidemiological parameters. Population-specific host population size is equal to human population size (Text S1). Basic epidemiological parameters are based on empirical estimates from H1N1: the duration of infection was chosen as 3 days [31] and the basic reproductive number (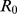) or average number of secondary infections arising from a primary infector during their infectious period in a completely susceptible population was chosen as 1.3 [31]. This results in a transmission rate 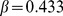. Although estimates of 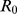 for pandemic H1N1 vary across studies, the exact 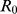 value is unlikely to affect the comparative simulations we perform as this is expected to equally impact the overall expansion rate and not the relative migration dynamics across populations. Force of infection 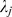 within population 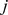 scales with infected frequency across populations following 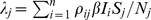, where the coupling coefficient 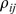 represents the rate of contacts from population *i* to population *j* relative to within-population contacts and 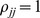. Other pairwise coupling coefficients are taken to be proportional to pairwise migration estimates, so that 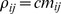, where 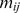 is the air travel based or phylogenetically estimated rate of migration from population *i* to population *j* per year and parameter *c* is fitted to the data. Parameter *c* is the only free parameter in this model and we set this to the value that maximizes correspondence between simulations and observations (see below). This ensures that we can use phylogeographic migration rates as *per capita* migration rates in the simulation model, despite their different scales. Compartments are updated according to a 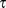-leaping algorithm [32] with one-day intervals.

Migration rates between populations in the SIR model are defined according to four scenarios, as follows: (A) equal rates, (B) rates proportional to the amount of air travel occurring between them (in terms of the number of passengers moving from one population to another), (C) rates proportional to Markov jump estimates based on a standard phylogeographic model (undertaken with and without BSSVS to reduce the number of rate parameters) and (D) a GLM model that only considers air travel as a predictor. To compare the spread of influenza under these simulated models to recorded H1N1 pandemic spread, we measure the relative correspondence between the mean peak times (across 100 simulations) and the observed peak times for all locations except Mexico (based on World Health Organization data; Text S1). Correspondence was measured using the Spearman's rank correlation coefficient, and tested with associated 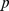-values obtained using a permutation test (Text S1), as well as using the mean average error (MAE; in days). We consider the Spearman's rank correlation coefficients to be more appropriate for our comparison because they are more robust to outliers, which are clearly present in the observed peaks. Therefore, the scaling of between-population coupling *c* for the various migration matrices was also adjusted so as to maximize Spearman's rank correlation.

## Results

### Air travel governs the global spatial spread of seasonal H3N2

**Figure 1:**
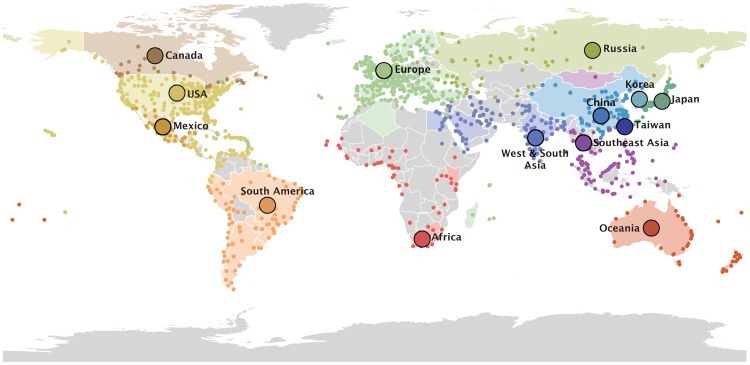
14 global air communities identified through a modularity maximization analyses of air transportation data. The colored dots represent the airports in each community for which passenger flux data was used in the analysis. The areas with corresponding colors represent the geographical area within the communities for which H3N2 sequence samples were available. The 14 communities and associated data are listed in Text S1.

To identify key factors in the seasonal dispersal of human influenza viruses, we use a Bayesian model selection procedure to estimate the phylogeographic history of H3N2 viruses sampled worldwide between 2002 and 2007 (Text S1), while concurrently evaluating the contribution of several potential predictors of spatial spread. In addition to considering two geographic discretizations of the available data, we also identify community structure in global air travel by determining partitions with high intra-community connectivity and low inter-community connectivity (Methods). Although this approach is blind to the airports' geographic locations, the 14 resulting global air communities are spatially compact with few exceptions (Fig. 1). We find air communities that are largely specific to Oceania, China, Japan, Sub-Saharan Africa, Mexico and Canada. Madagascar, Réunion and some Caribbean destinations are examples of exceptions that are, as non-European locations, connected to a European air community.

**Figure 2:**
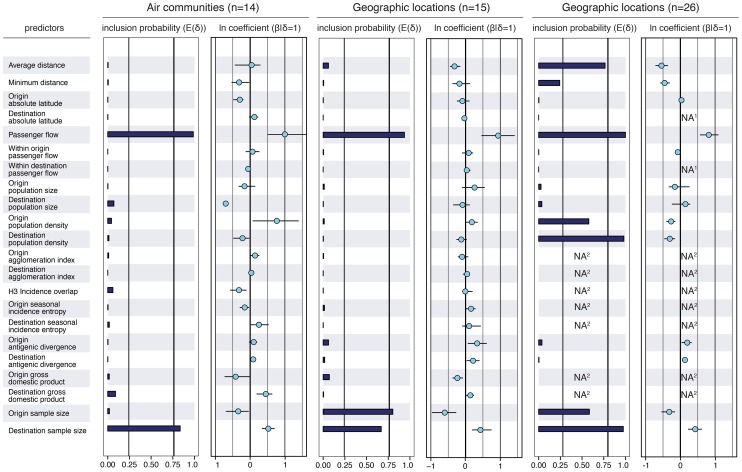
Predictors of global H3N2 diffusion among the 14 air communities and the 15 & 26 geographic locations. The inclusion probabilities are defined by the indicator expectations 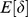 because they reflect the frequency at which the predictor is included in the model and therefore represent the support for the predictor. Indicator expectations corresponding to Bayes factor support values of 10 and 100 are represented by a thin and thick vertical line respectively in these bar plots. The contribution of each predictor, when included in the model (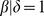), where 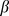 is the coefficient or effect size, is represented by the mean and credible intervals of the GLM coefficients on a log scale. NA^1^: no conditional effect size available because the predictor was never included in the model. We tested different population size and density measures, different incidence-based measures and different seasonal measures (Text S1), but only list the estimates for a representative predictor for the sake of clarity. The estimates for the full set of predictors are summarized for each sub-sampled data set in Fig. S5. NA^2^: no indicator expectation or conditional effect size available because the predictor was not available for this discretization of the sequence data.

Our analysis reveals that many potential predictors of global influenza virus spread are not associated with viral lineage movement, specifically, geographical proximity, demography and economic measures, antigenic divergence, epidemiological synchronity and seasonality do not yield noticeable support (Fig. 2). Instead, we find consistent and strong evidence that air passenger flow is the dominant driver of the global dissemination of H3N2 influenza viruses. This is reflected in both the estimated size of the effect of this variable (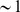 on a log scale) and the statistical support for its inclusion in the model (posterior probability >0.93 and Bayes factor >760). This effect size means that viral lineage movement rates are about 15 times higher for connections with the highest passenger flow compared to connections with the lowest flow, controlling for all other predictors. The result is robust when we repeat the analysis (i) using different partitions of sampling locations (air communities and different geographic partitions, Fig. 2), (ii) using different sequence sub-samples for the air communities (Fig. S1), (iii) using the full data set or a small but more balanced number of sub-samples (Fig. S2), and (iv) using a more liberal prior specification on predictor inclusion (Fig. S3). We down-sampled particular air communities or geographic regions relative to their population sizes (Text S1), which still leaves considerable heterogeneity in sample sizes, explaining why they are included as an explanatory variable in the GLM model. Our aim is not to demonstrate a role for sample sizes in phylogeography, but by explicitly including them as predictive variables, we raise the credibility that other predictors are not included in the model because of sampling bias. We note that the sample size predictors may in fact absorb some of the effect of air travel because a GLM model that only considers passenger flux as a predictor of H3N2 movement among the air communities results in a higher mean effect of size of about 1.5.

To also explore spatial dynamics at smaller scales, we further partition large geographical regions that are administratively coherent, such as the USA, China, Japan and Australia, resulting in 26 global sampling regions (Text S1). In this analysis, air travel again predicts viral movement (posterior probability >0.99 and Bayes factor >18000), but the movement is also inversely associated with geographical distance between locations (posterior probability = 0.76 and Bayes factor = 87), and, less intuitively, with origin and destination population densities (although the size of the latter effects are weaker, Fig. 2). The negative association of population density with viral movement may suggest that commuting is less likely, *per capita*, to occur out of, or into, dense subpopulations.

### Unravelling source-sink dynamics

**Figure 3:**
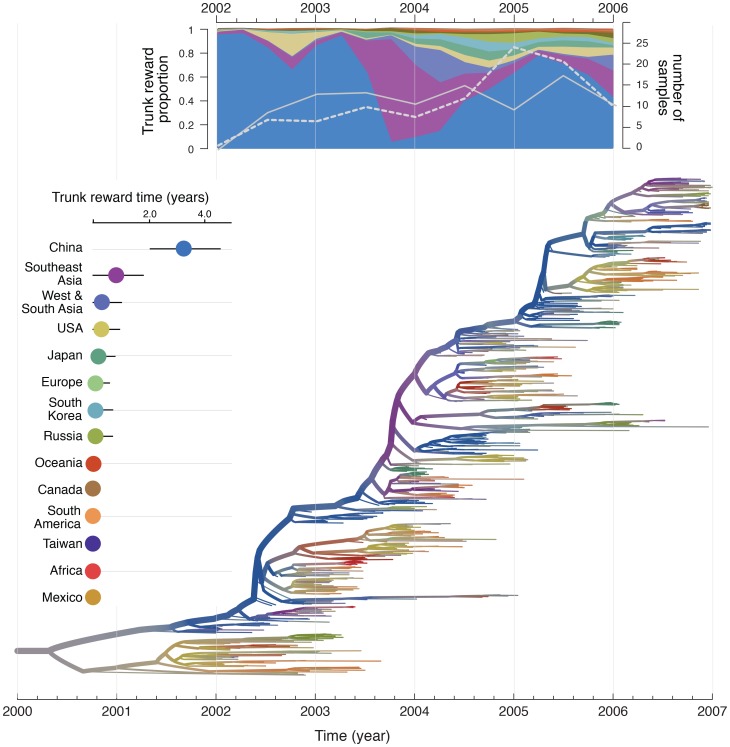
Phylogeographic reconstruction and spatial history of the trunk lineage. Maximum clade credibility (MCC) tree colored according to the time spent in the air communities as inferred by the GLM diffusion model. The tree represents one of the three different sub-sampled data sets discretized according to the 14 air communities. Branches are colored according the Markov reward estimates for each location. The uncertainty of these estimates is represented by superimposing an additional gray color proportional to the Shannon entropy of the Markov reward values. The trunk lineage in the tree is represented by the thick upper branch path from the root to the nodes that represent the ancestors of samples that are exclusively from December 2006. The total time spent in each location (in years) along the trunk between 2002 and 2006 is plotted on the left of the tree. The trunk reward proportion for each location through time between 2002 and 2006 is summarized at the top of the tree. Both the total trunk time and the trunk reward proportions through time are averaged over the three sub-sampled data sets. In the trunk proportion through time plot, the number of Southeast Asian and Chinese samples are represented by a white full and dashed line respectively (secondary Y-axis).

Although not the main focus of the current study, our integrated approach also provides phylogeographic reconstructions that offer insights into the global source-sink dynamics of human influenza. The trunk or backbone of phylogenies reconstructed from temporally-sampled hemagglutinin genes (Fig. 3) represents the lineage that successfully persists from one epidemic year to the next [14], [33]. We determine the spatial history of this lineage using Markov rewards in the posterior tree distribution, thereby estimating the contribution of each location to the persistence of the trunk lineage from 2002 to 2006 (Fig. 3). These estimates provide strong support for mainland China as the principal H3N2 source population, occupying close to 60% of the trunk time in the H3N2 phylogenies (Fig. 3), followed by Southeast Asia, which comprises about 15% of the trunk time. We further examine temporal heterogeneity in the source-sink process by combining a summary of the estimated trunk location through time together with an phylogenetic summary in Fig. 3, which suggests that the above-mentioned proportions arose from the presence of the trunk lineage in China during 2002 to mid 2003 and late 2004 to 2006, interrupted by a period when the virus appeared to have a Southeast Asian H3N2 source. However, we cannot rule out the impact of temporal sampling heterogeneity on these estimates because the Southeast Asian trunk dominance precedes a period of higher sampling availability for Southeast Asia relative to mainland China (Fig. 3). The important role of mainland China in seeding the global seasonal spread of human influenza results in a high net migration out of this air community (Fig. S4). However, air communities that do not contribute significantly to the trunk can also maintain high net outflow, in particular the USA, which may be seeded by relatively few introductions each year whilst exporting comparatively more viruses to other locations during the epidemic season.

### Viral evolutionary history combined with human mobility predicts the pandemic spread of H1N1

**Figure 4:**
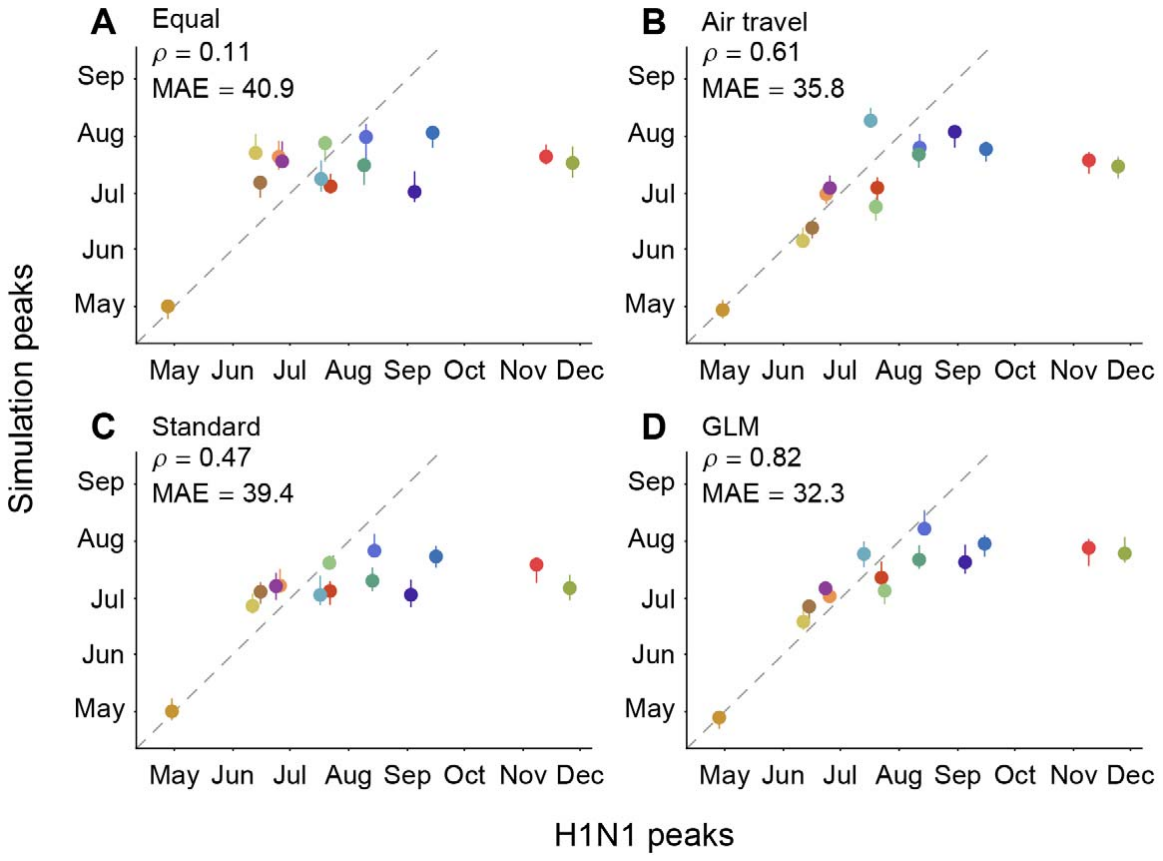
Correlation among observed H1N1 peaks and simulated peaks based on different migration rate models. The simulations were performed using (A) an equal rate matrix, (B) a matrix of airline passengers flows, (C) standard phylogeographic estimates and (D) GLM phylogeographic estimates only considering air travel as a predictor. Spearman rank correlations (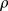) and mean absolute error (MAE; in days) considering all locations except for Mexico are provided for each comparison. The data points are colored according to the air communities represented in Fig. 1. The dotted lines represent a 1-to-1 correspondence between observed peaks and simulated H1N1 peaks.

In order to assess the extent to which evolutionary analyses such as ours benefit from integrating host mobility data, we examine their predictive performance by using them to predict the relative timing of the geographic spread of the pandemic H1N1 influenza variant that emerged in 2009. We conduct simulations of the spread of a novel pathogen out of Mexico using an SIR model whose transmission parameters are informed by epidemiological estimates obtained for pandemic H1N1 [31] and whose spatial spread is determined by one of four different migration rate models, each defined by a different matrix of movement rates among all pairs of locations (Methods). We measure the relative correspondence between the simulated and observed H1N1 peaks for each location except Mexico using a Spearman's rank correlation coefficient (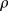) and mean absolute error (MAE; in days)(Fig. 4).

An equal rates model (A), which does not express any migration rate preference, results in a weak match (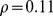, *P* = 0.73, MAE = 40.9 days) between the simulations and the observed spatial spread of H1N1 (Fig. 4), indicating that the population sizes included in the SIR model for each region offer limited predictive performance. As expected, adding information on the number of airline passengers (model B) yields a large improvement in correspondence between simulations and observations (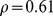, *P* = 0.03, MAE = 35.8 days). In contrast, a standard parameter-rich phylogeographic model that is only informed by sequence data and not air traffic information (model C) yields only part of this improvement in predictive performance (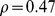, *P* = 0.10, MAE = 39.4 days). However, if inference under model C is made more efficient by focusing on a small set of parameters (using BSSVS, [21]; see Methods) then phylogeographic estimates yield a predictive performance (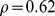, *P* = 0.02, MAE = 36.4 days, Fig. S5) that is close to that of the air travel model (B). Finally, the GLM model (D) predicts the observed spread of H1N1 more accurately than all other models (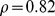, *P*<0.01, MAE = 32.3), suggesting that global influenza transmission is best predicted by combining passenger flux data with the information on viral lineage movement contained in sequence data. The simulations generally correspond better with observed H1N1 peaks during the initial period of pandemic expansion, while the epidemic peaks for Russia and Africa occur significantly earlier in the simulations than in reality. This is likely due to the multi-peaked character of the regional epidemics (Text S1); the H1N1 virus spreads to most of the world during the first pandemic wave, whereas regions like Russia and Africa appeared to miss the first wave entirely. Seasonal effects that are unaccounted for by our simulation may at least partly explain the outliers, but they affect the models we aim to compare in a very similar way. Because of the outliers, we consider the non-parametric Spearman's 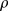 to be a more appropriate measure of correspondence than the MAE, but they are consistent in their model ranking. We note that absolute prediction errors can be considerably improved by only considering the 9 air communities that peaked prior to September, 2009, which returns a MAE of 11.2 day for the GLM model. However, because of the difficulties in establishing initial waves and their peaks, and the uncertainty in our epidemiological model, we caution against more detailed interpretation of these simulations beyond the general trends we extract here.

## Discussion

The prevention and control of influenza at the global scale relies critically on our understanding of its mode of geographical dissemination. Here, we demonstrate that such dynamics are most powerfully investigated by combining phylogeographic history with empirical data on the patterns of human movement worldwide. Our analysis strongly suggests that air travel is key to global influenza spread, an intuitive result that has long been predicted by modeling studies (e.g. [5]), but has, until now, remained difficult to obtain from empirical data. The dominant predictors of influenza spread will undoubtedly be scale-dependent, as indicated here by the importance of geographic distance as a predictor within more confined geographic areas (Fig. 2), which may represent forms of human mobility other than air travel, such as workplace commuting [9]. This indicates that our statistical framework could also prove valuable in testing hypotheses at smaller scales, where the underlying spatial processes may be less obvious, provided adequate sequence and empirical movement data are available. One of the limitations of the current heterogeneous sampling of H3N2 sequences worldwide is that geographic partitions need to be adjusted to account for the number of samples per location, which results in regions of widely different areas and population sizes. More representative sampling across the globe, or within a more geographically confined area of interest, will allow for more appropriate geographic partitioning and may facilitate more detailed spatial hypothesis testing based on the associated demographic and mobility measures. In particular, if sequences were sampled appropriately then our inference method could incorporate the rich geographic data that is currently available as global gridded population data sets [34]. In addition, many of the predictors used here can be improved in accuracy and resolution, for example by accounting for seat occupancy and actual origin-destination flows in air traffic passenger fluxes.

Due to the difficulties associated with geographic partitioning, we used algorithms to optimally define communities in the global air transportation network as an alternative strategy to specify phylogeographic states, and subsequently show that our GLM results are robust to the different partitions used. Because air travel is a consistent and highly supported explanatory variable for global influenza dispersal, communities within the air transportation network are likely to provide the most appropriate spatial structuring of our data. However, in addition to the partitioning itself, further research is also needed to select the appropriate number of samples from the resulting regions to improve on *ad hoc* down-sampling based on population size.

Although identifying the causes of pathogen spread is of great importance in spatial epidemiology, integrating this information in evolutionary models also offers major advantages for phylogeographic reconstructions and their relevance to infectious disease surveillance and pandemic preparedness. By capturing a more realistic process of spatial spread, our novel approach results in more credible reconstructions of spatial evolutionary history, which may shed further light on the persistence and migration dynamics of human influenza viruses. Because of the importance of influenza dynamics for vaccine strain selection, different phylogeographic reconstructions have attempted to characterize the global population structure of the virus and have arrived at somewhat mixed findings [3], [14], [17]. This may be explained by the use of both different sampling and different methodology. The data and methods used here corroborate the explorations of antigenic and genetic divergence by [3] and demonstrate the prominence of mainland China and Southeast Asia as locations of trunk lineage persistence. Our findings are however based on roughly the same genetic data, and our approach of inferring the spatial history of the trunk lineage through Markov reward estimates may be viewed as the more direct, statistical equivalent of measuring strain location distance from the trunk [3]. Although we find a strong signal for the presence of the trunk lineage in mainland China and Southeast Asia, our analysis is restricted to the period 2002 to 2006, and thus we make no conclusions about the location of the trunk lineage outside of this period. The degree of temporal stochasticity in the source location of seasonal influenza and its heterogeneity among different influenza variants has yet to be determined and requires datasets of longer duration. Moreover, we suggest that analyses of future data sets that are more comprehensively sampled through time will also benefit from phylogeographic models that can accommodate temporal heterogeneity in movement rates. Such models may also improve the performance of some explanatory variables. For example, in the analysis presented here, we do not consider the absence of support for seasonality as a predictor in our GLM model as evidence against seasonality in H3N2 spread. Rather, it simply reflects the difficulty in incorporating seasonality into a time-homogeneous model of lineage movement. Developments are now underway to appropriately accommodate heterogeneity in spatial spread through time.

By using models to predict the observed global emergence of pandemic H1N1, we demonstrate that an approach that integrates passenger flux data with viral genetic data provides a more accurate prediction of global epidemic spread than those which include only one source of information. Although the prediction improvement of the combined data over the passenger flux data alone is not very large, it remains remarkable because we attempt to predict the spatial expansion of an epidemic lineage (pandemic H1N1) from the seasonal dynamics of another lineage (H3N2) and because the main process underlying the global dispersal of H3N2 influenza appears to be air travel itself. Passenger flux data among pairs of locations is symmetric, thus it is possible that the phylogeographic data is capable of capturing asymmetry in the seasonal process of viral spread, which may also be important in explaining the spatial expansion of pandemic H1N1. Investigations using more advanced simulation techniques, e.g. [35], may be able to build upon the conceptual bridge between genetic data and epidemiological modeling implied by our findings. Future prediction efforts may also need to focus on alternative scenarios of spatial spread, as highlighted by the recent emergence of a novel avian influenza H7N9 lineage in China [36]. Should this virus evolve sustained human-to-human transmissibility, then airline-passenger data and flight routes from the outbreak regions in particular, would be able to pinpoint worldwide regions of immediate risk. If the virus remains restricted to avian hosts, however, risk maps for the transmission of avian influenza viruses (perhaps based on predictors calibrated against H5N1 avian influenza) may help to target H7N9 surveillance and control efforts. In conclusion, our framework is applicable to different infectious diseases and provides new opportunities for explicitly testing how host behavior and ecology shapes the spatial distribution of pathogen genetic diversity.

## Supporting Information

Dataset S1 **XML example for running the GLM-diffusion model in BEAST and associated empirical trees file.** The XML file, airCommunitiesMM_1.xml, specifies the data for one of the air community subsets as well as the model and MCMC settings. The empirical trees file required to run the analysis, subset1.trees, contains a sample of 500 trees from the posterior distribution of the sequence analysis.
(ZIP)

Figure S1
**Predictors of global H3N2 diffusion among the 14 air communities for three different sub-samples of the sequence data.** Each combination of inclusion probability bar plot and corresponding coefficient plot represents the GLM results for one of the three different sub-samples of the H3N2 sequence data. These sub-samples were obtained by randomly down-sampling the four locations with the highest number of samples relative to their population size for each sampling year. The inclusion probabilities are defined by the indicator expectations 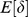 because they reflect the frequency at which the predictor is included in the model and therefore represent the support for the predictor. Indicator expectations corresponding to Bayes factor support values of 10 and 100 are represented by a thin and thick vertical line respectively in these bar plots. The contribution of each predictor, when included in the model (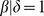), where 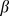 is the coefficient or effect size, is represented by the mean and credible intervals of the GLM coefficients on a log scale. If the inclusion probability is zero for a predictor, no corresponding GLM coefficient is shown. We tested different population size and density measures, different incidence-based measures and different seasonal measures (Text S1), but only list the estimates for a representative predictor for the sake of clarity.(PDF)

Figure S2
**Predictors of global H3N2 diffusion among the 14 air communities for the full data set and for two different sub-samples with a balanced number of sequences per location.** Each combination of inclusion probability bar plot and corresponding coefficient plot represents the GLM results for the full data set (A) and the two different sub-samples (B and C) of the H3N2 sequence data. These sub-samples were obtained by randomly down-sampling 25 sequences from locations for which the number samples available exceeded that number. The inclusion probabilities are defined by the indicator expectations 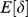 because they reflect the frequency at which the predictor is included in the model and therefore represent the support for the predictor. Indicator expectations corresponding to Bayes factor support values of 10 and 100 are represented by a thin and thick vertical line respectively in these bar plots. The contribution of each predictor, when included in the model (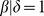), where 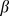 is the coefficient or effect size, is represented by the mean and credible intervals of the GLM coefficients on a log scale. If the inclusion probability is zero for a predictor, no corresponding GLM coefficient is shown. We tested different population size and density measures, different incidence-based measures and different seasonal measures (Text S1), but only list the estimates for a representative predictor for the sake of clarity.(PDF)

Figure S3
**Predictors of global H3N2 diffusion among the 14 air communities and the 15 & 26 geographic locations using equal prior probability on the inclusion and exclusion of each predictor.** The inclusion probabilities are defined by the indicator expectations 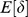 because they reflect the frequency at which the predictor is included in the model and therefore represent the support for the predictor. As opposed the analysis reported in main manuscript (Fig. 2), which specifies a prior probability of 0.019 on each predictor's inclusion, we here specify a prior probability of 0.5 on the inclusion of each predictor. Indicator expectations corresponding to Bayes factor support values of 3 and 20 are shown as a thin and thick vertical line respectively in these bar plots. The contribution of each predictor, when included in the model (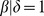), where 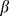 is the coefficient or effect size, is represented by the mean and credible intervals of the GLM coefficients on a log scale. NA^1^: no conditional effect size available because the effect was never included in the model. We tested different population size and density measures, different incidence-based measures and different seasonal measures (Text S1), but only list the estimates for a representative predictor for the sake of clarity. NA^2^: no indicator expectation or conditional effect size available because the predictor was not available for this discretization of the sequence data. A comparison with the analysis reported in main manuscript (Fig. 2) indicates that our results are robust to the prior specification for the inclusion probabilities; only the scale of the Bayes factor support shifts to lower values because of the higher prior odds (1∶1 as opposed to 0.019∶0.981) in this case.(PDF)

Figure S4 **Net Markov jump counts for the 14 air communities.** For each air community, we summarize the average net Markov jumps (jumps to - jumps from) and their 95% credible intervals. The estimates are ordered from the lowest (top; jumps to <jumps from) to highest (bottom; jumps to >jumps from) net jumps. The data points are colored according to the air communities represented in Fig. 1 in the main text.
(PDF)

Figure S5
**Correlation among observed H1N1 peaks and simulated peaks based on the BSSVS estimates.** The Spearman rank correlation (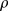) and mean absolute error (MAE; in days) for all locations except for Mexico is shown at the top left. The data points are colored according to the air communities represented in Fig. 1 in the main text.(PDF)

Text S1 **Additional materials & methods information and evaluation of the GLM-diffusion approach on empirical data.** This supporting information text describes additional information on the following topics: (i) sequence and location data, (ii) incorporating uncertainty in air community assignment, (iii) Bayesian statistical analysis of sequence and trait evolution and (iv) comparing simulated spatial expansion to recorded H1N1 pandemic data. In addition, we report on an evaluation of the GLM-diffusion approach on empirical data. The supporting information text refers to figures and tables included in this text as Fig. S1, S2, S3, S4 or Table S1–S5 *in Text S1*, as well to the additional supporting information Figures S1, S2, S3, S4, S5.
(PDF)
