# Supplementary material for: Unifying Viral Genetics and Human Transportation Data to Predict the Global Transmission Dynamics of Human Influenza H3N2
Source: PLoS Pathog. 2014 Feb 20;10(2):e1003932. doi: 10.1371/journal.ppat.1003932 (PMC3930559; doi:10.1371/journal.ppat.1003932)
Supplement: Figure S1 — Predictors of global H3N2 diffusion among the 14 air communities for three different sub-samples of the sequence data. Each combination of inclusion probability bar plot and corresponding coefficient plot represents the GLM results for one of the three different sub-samples of the H3N2 sequence data. These sub-samples were obtained by randomly down-sampling the four locations with the highest number of samples relative to their population size for each sampling year. The inclusion probabilities are defined by the indicator expectations because they reflect the frequency at which the predictor is included in the model and therefore represent the support for the predictor. Indicator expectations corresponding to Bayes factor support values of 10 and 100 are represented by a thin and thick vertical line respectively in these bar plots. The contribution of each predictor, when included in the model (), where is the coefficient or effect size, is represented by the mean and credible intervals of the GLM coefficients on a log scale. If the inclusion probability is zero for a predictor, no corresponding GLM coefficient is shown. We tested different population size and density measures, different incidence-based measures and different seasonal measures (Text S1), but only list the estimates for a representative predictor for the sake of clarity. (PDF) [file ppat.1003932.s002.pdf]

Supplementary Figure S5

Predictors of global H3N2 diffusion among the 14 air communities for three different sub-samples of the sequence data. Each combination of inclusion probability bar plot and corresponding coefficient plot represents the GLM results for one of the three different sub-samples of the H3N2 sequence data. These sub-samples were obtained by randomly down-sampling the four locations with the highest number of samples relative to their population size for each sampling year. The inclusion probabilities are defined by the indicator expectations $E[\delta]$ because they reflect the frequency at which the predictor is included in the model and therefore represent the support for the predictor. Indicator expectations corresponding to Bayes factor support values of 10 and 100 are represented by a thin and thick vertical line respectively in these bar plots. The contribution of each predictor, when included in the model ($\beta|\delta = 1$), where β is the coefficient or effect size, is represented by the mean and credible intervals of the GLM coefficients on a log scale. We tested different population size and density measures, different incidence-based measures and different seasonal measures (*Supplementary Text S1*), but only list the estimates for a representative predictor for the sake of clarity.

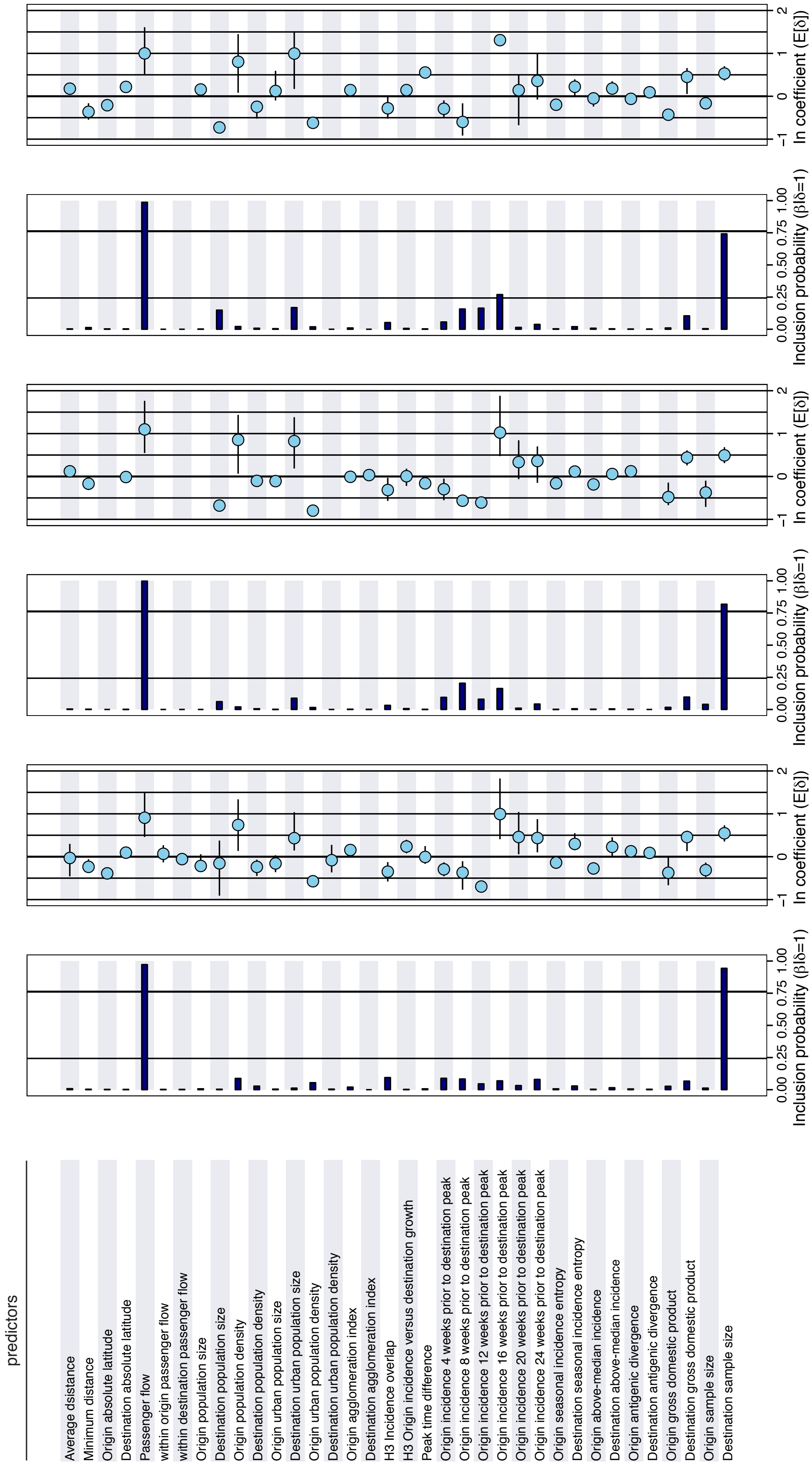
predictors
Average dsistance
Minimum distance
Origin absolute latitude
Destination absolute latitude
Passenger flow
within origin passenger flow
within destination passenger flow
Origin population size
Destination population size
Origin population density
Destination population density
Origin urban population size
Destination urban population size
Origin urban population density
Destination urban population density
Origin agglomeration index
Destination agglomeration index
H3 Incidence overlap
H3 Origin incidence versus destination growth
Peak time difference
Origin incidence 4 weeks prior to destination peak
Origin incidence 8 weeks prior to destination peak
Origin incidence 12 weeks prior to destination peak
Origin incidence 16 weeks prior to destination peak
Origin incidence 20 weeks prior to destination peak
Origin incidence 24 weeks prior to destination peak
Origin seasonal incidence entropy
Destination seasonal incidence entropy
Origin above-median incidence
Destination above-median incidence
Origin antigenic divergence
Destination antigenic divergence
Origin gross domestic product
Destination gross domestic product
Origin sample size
Destination sample size
Inclusion probability (β|δ=1)
0.00 0.25 0.50 0.75 1.00
ln coefficient (E[δ])
−1 0 1 2
