# Supplementary figures and images for: Unifying Viral Genetics and Human Transportation Data to Predict the Global Transmission Dynamics of Human Influenza H3N2
Source: PLoS Pathog. 2014 Feb 20;10(2):e1003932. doi: 10.1371/journal.ppat.1003932 (PMC3930559; doi:10.1371/journal.ppat.1003932)

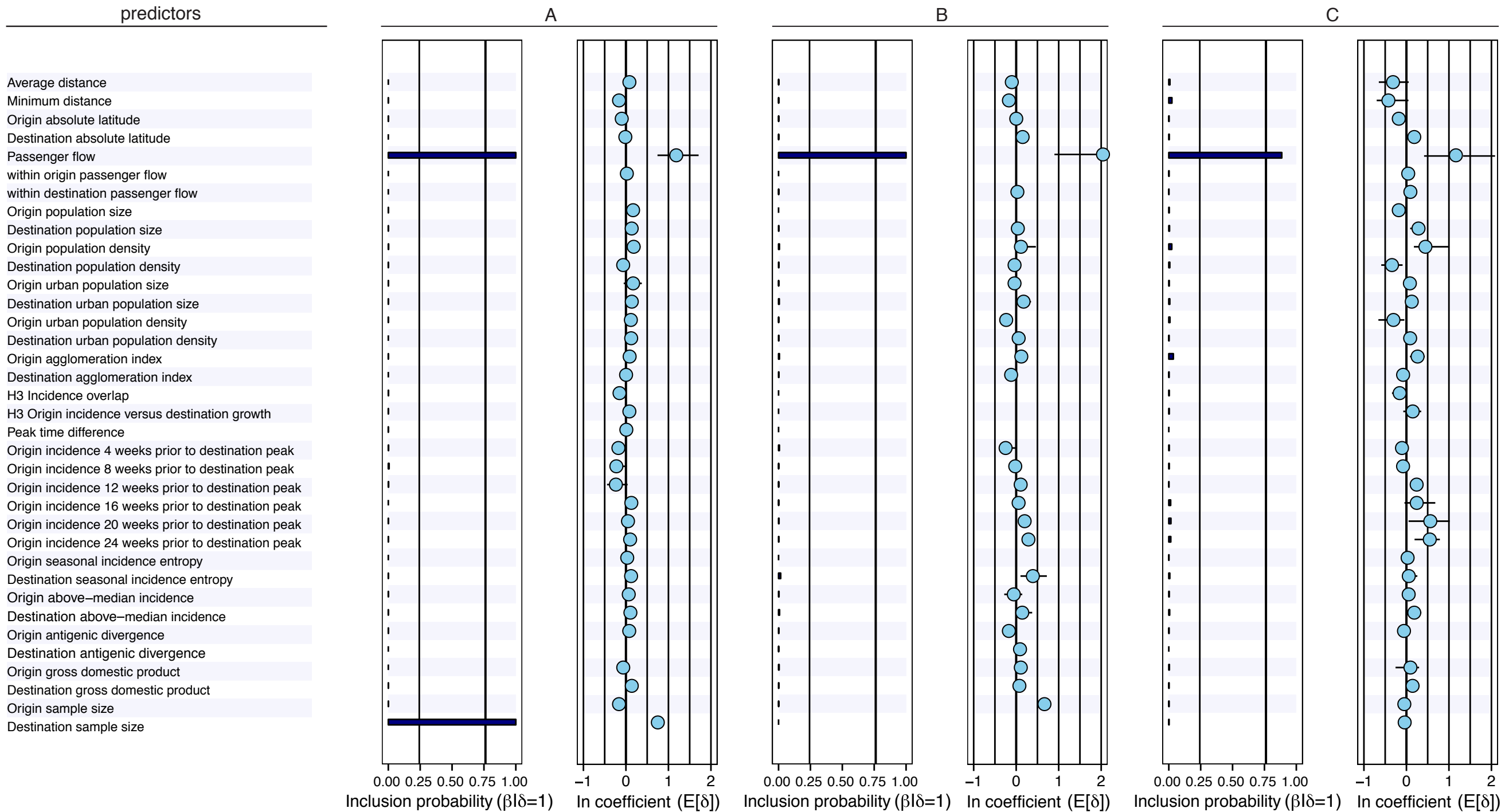

Supplement: Figure S2 — Predictors of global H3N2 diffusion among the 14 air communities for the full data set and for two different sub-samples with a balanced number of sequences per location. Each combination of inclusion probability bar plot and corresponding coefficient plot represents the GLM results for the full data set (A) and the two different sub-samples (B and C) of the H3N2 sequence data. These sub-samples were obtained by randomly down-sampling 25 sequences from locations for which the number samples available exceeded that number. The inclusion probabilities are defined by the indicator expectations because they reflect the frequency at which the predictor is included in the model and therefore represent the support for the predictor. Indicator expectations corresponding to Bayes factor support values of 10 and 100 are represented by a thin and thick vertical line respectively in these bar plots. The contribution of each predictor, when included in the model (), where is the coefficient or effect size, is represented by the mean and credible intervals of the GLM coefficients on a log scale. If the inclusion probability is zero for a predictor, no corresponding GLM coefficient is shown. We tested different population size and density measures, different incidence-based measures and different seasonal measures (Text S1), but only list the estimates for a representative predictor for the sake of clarity. (PDF) [file ppat.1003932.s003.pdf]
