# Supplementary material for: Unifying Viral Genetics and Human Transportation Data to Predict the Global Transmission Dynamics of Human Influenza H3N2
Source: PLoS Pathog. 2014 Feb 20;10(2):e1003932. doi: 10.1371/journal.ppat.1003932 (PMC3930559; doi:10.1371/journal.ppat.1003932)
Supplement: Figure S3 — Predictors of global H3N2 diffusion among the 14 air communities and the 15 & 26 geographic locations using equal prior probability on the inclusion and exclusion of each predictor. The inclusion probabilities are defined by the indicator expectations because they reflect the frequency at which the predictor is included in the model and therefore represent the support for the predictor. As opposed the analysis reported in main manuscript (Fig. 2), which specifies a prior probability of 0.019 on each predictor's inclusion, we here specify a prior probability of 0.5 on the inclusion of each predictor. Indicator expectations corresponding to Bayes factor support values of 3 and 20 are shown as a thin and thick vertical line respectively in these bar plots. The contribution of each predictor, when included in the model (), where is the coefficient or effect size, is represented by the mean and credible intervals of the GLM coefficients on a log scale. NA1: no conditional effect size available because the effect was never included in the model. We tested different population size and density measures, different incidence-based measures and different seasonal measures (Text S1), but only list the estimates for a representative predictor for the sake of clarity. NA2: no indicator expectation or conditional effect size available because the predictor was not available for this discretization of the sequence data. A comparison with the analysis reported in main manuscript (Fig. 2) indicates that our results are robust to the prior specification for the inclusion probabilities; only the scale of the Bayes factor support shifts to lower values because of the higher prior odds (1∶1 as opposed to 0.019∶0.981) in this case. (PDF) [file ppat.1003932.s004.pdf]

Supplementary Figure S7

Predictors of global H3N2 diffusion among the 14 air communities and the 15 & 26 geographic locations using equal prior probability on the inclusion and exclusion of each predictor. The inclusion probabilities are defined by the indicator expectations $E[\delta]$ because they reflect the frequency at which the predictor is included in the model and therefore represent the support for the predictor. As opposed the analysis reported in main manuscript (Fig. 2), which specifies a prior probability of 0.019 on each predictor's inclusion, we here specify a prior probability of 0.5 on the inclusion of each predictor. Indicator expectations corresponding to Bayes factor support values of 3 and 20 are shown as a thin and thick vertical line respectively in these bar plots. The contribution of each predictor, when included in the model ($\beta|\delta = 1$), where β is the coefficient or effect size, is represented by the mean and credible intervals of the GLM coefficients on a log scale. NA^1: no conditional effect size available because the effect was never included in the model. We tested different population size and density measures, different incidence-based measures and different seasonal measures (*Supplementary Text S1*), but only list the estimates for a representative predictor for the sake of clarity. NA^2: no indicator expectation or conditional effect size available because the predictor was not available for this discretization of the sequence data. A comparison with the analysis reported in main manuscript (Fig. 2) indicates that our results are robust to the prior specification for the inclusion probabilities; only the scale of the Bayes factor support shifts to lower values because of the higher prior odds (1:1 as opposed to 0.019:0.981) in this case.

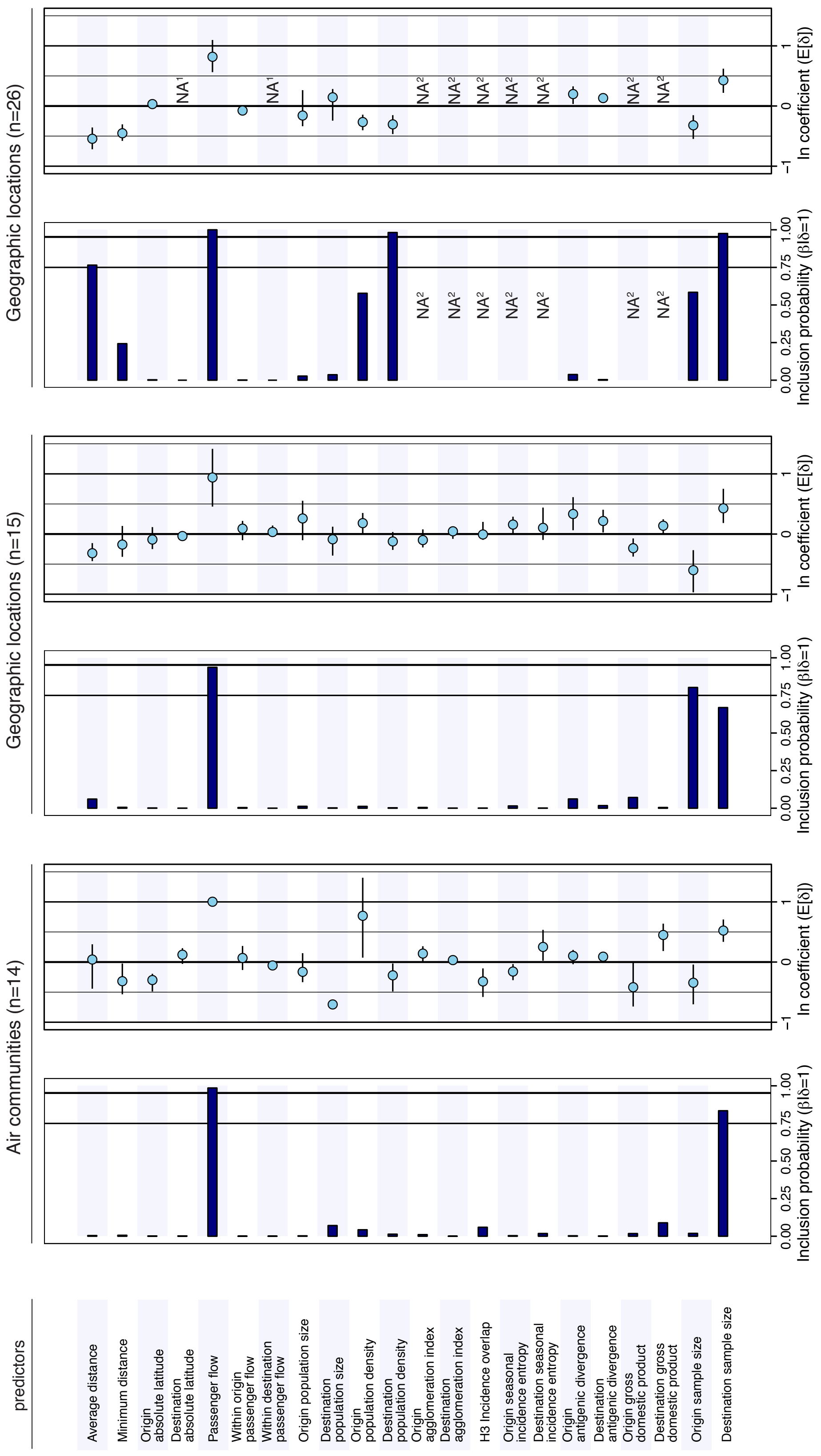

predictors
Air communities (n=14)
Geographic locations (n=15)
Geographic locations (n=26)
Inclusion probability (β|δ=1)
ln coefficient (E[δ])
0.00
0.25
0.50
0.75
1.00
−1
0
1
NA¹
NA²
Average distance
Minimum distance
Origin absolute latitude
Destination absolute latitude
Passenger flow
Within origin passenger flow
Within destination passenger flow
Origin population size
Destination population size
Origin population density
Destination population density
Origin agglomeration index
Destination agglomeration index
H3 Incidence overlap
Origin seasonal incidence entropy
Destination seasonal incidence entropy
Origin antigenic divergence
Destination antigenic divergence
Origin gross domestic product
Destination gross domestic product
Origin sample size
Destination sample size
