# Supplementary material for: Unifying Viral Genetics and Human Transportation Data to Predict the Global Transmission Dynamics of Human Influenza H3N2
Source: PLoS Pathog. 2014 Feb 20;10(2):e1003932. doi: 10.1371/journal.ppat.1003932 (PMC3930559; doi:10.1371/journal.ppat.1003932)
Supplement: Figure S4 — Net Markov jump counts for the 14 air communities. For each air community, we summarize the average net Markov jumps (jumps to - jumps from) and their 95% credible intervals. The estimates are ordered from the lowest (top; jumps to jumps from) net jumps. The data points are colored according to the air communities represented in Fig. 1 in the main text. (PDF) [file ppat.1003932.s005.pdf]

Supplementary Figure S8

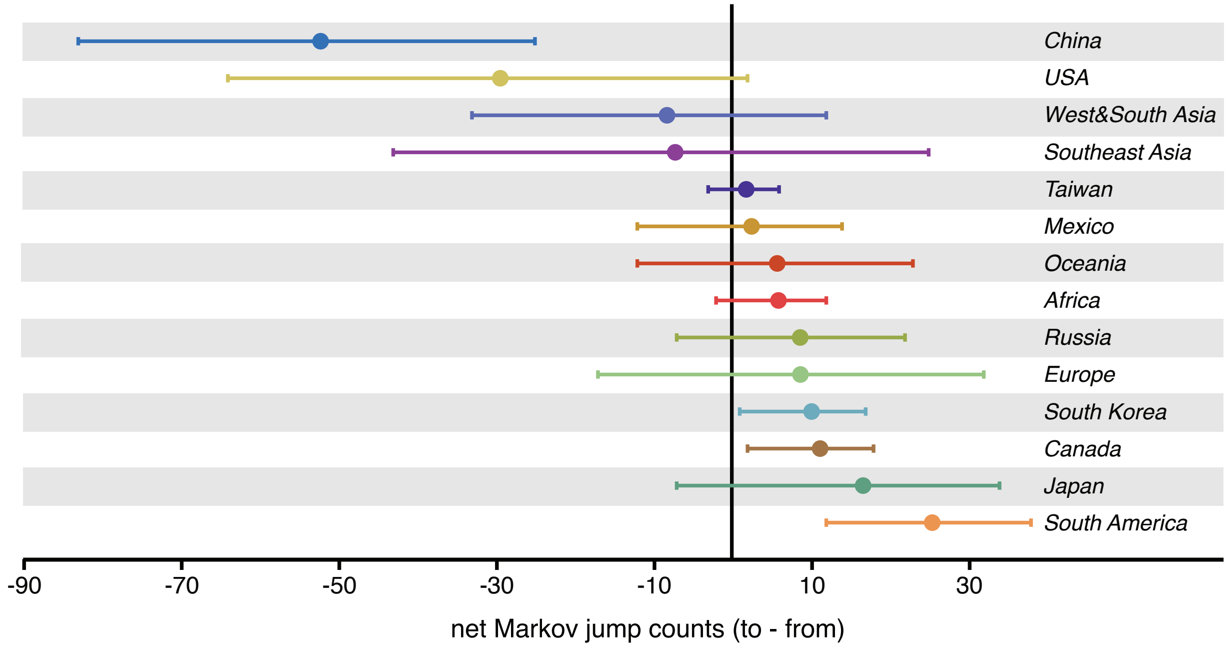

Figure S8: **Net Markov jump counts for the 14 air communities.** For each air community, we summarize the average net Markov jumps (jumps to - jumps from) and their 95% credible intervals. The estimates are ordered from the lowest (top, jumps to < jumps from) to highest (bottom, jumps to > jumps from) net jumps. The data points are colored according to the air communities represented in Fig. 1 in the main text.
