# Supplementary material for: Unifying Viral Genetics and Human Transportation Data to Predict the Global Transmission Dynamics of Human Influenza H3N2
Source: PLoS Pathog. 2014 Feb 20;10(2):e1003932. doi: 10.1371/journal.ppat.1003932 (PMC3930559; doi:10.1371/journal.ppat.1003932)
Supplement: Figure S5 — Correlation among observed H1N1 peaks and simulated peaks based on the BSSVS estimates. The Spearman rank correlation () and mean absolute error (MAE; in days) for all locations except for Mexico is shown at the top left. The data points are colored according to the air communities represented in Fig. 1 in the main text. (PDF) [file ppat.1003932.s006.pdf]

Supplementary Figure S9

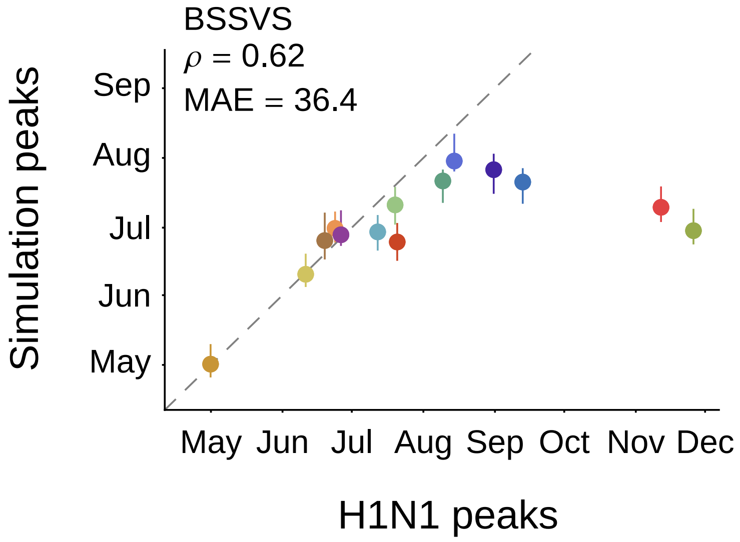

Figure S9: **Correlation among observed H1N1 peaks and simulated peaks based on the BSSVS estimates.** The Spearman rank correlation (ρ) and mean absolute error (MAE; in days) for all locations except for Mexico is shown at the top left. The data points are colored according to the air communities represented in Fig. 1 in the main text.
