# Supplementary material for: Unifying Viral Genetics and Human Transportation Data to Predict the Global Transmission Dynamics of Human Influenza H3N2
Source: PLoS Pathog. 2014 Feb 20;10(2):e1003932. doi: 10.1371/journal.ppat.1003932 (PMC3930559; doi:10.1371/journal.ppat.1003932)
Supplement: Text S1 — Additional materials & methods information and evaluation of the GLM-diffusion approach on empirical data. This supporting information text describes additional information on the following topics: (i) sequence and location data, (ii) incorporating uncertainty in air community assignment, (iii) Bayesian statistical analysis of sequence and trait evolution and (iv) comparing simulated spatial expansion to recorded H1N1 pandemic data. In addition, we report on an evaluation of the GLM-diffusion approach on empirical data. The supporting information text refers to figures and tables included in this text as Fig. S1, S2, S3, S4 or Table S1–S5 in Text S1, as well to the additional supporting information Figures S1, S2, S3, S4, S5. (PDF) [file ppat.1003932.s007.pdf]

Supporting information Text S1

for

Unifying Viral Genetics and Human Transportation Data to Predict the Global Transmission Dynamics of Human Influenza H3N2

Contents

1 Materials and Methods 1
1.1 Sequence and location data 1
1.2 Incorporating uncertainty in air community assignment 3
1.3 Bayesian statistical analysis of sequence and trait evolution 4
1.3.1 GLM diffusion implementation and predictor support 5
1.3.2 GLM diffusion predictors 6
1.3.3 Fitting diffusion models to empirical tree distributions 10
1.4 Comparing simulated spatial expansion to recorded H1N1 pandemic data . . 11

2 Evaluation of the GLM-diffusion approach on empirical data 12

4 References 15

1 Materials and Methods

1.1 Sequence and location data

We compiled 1441 hemagglutinin sequences with known date and location of sampling previously obtained by [1]. These sequences were sampled globally from 2002 to 2007 and are representative of a larger sampling (13,000 isolates) used for antigenic analysis [1]. We explored different spatial and air travel-assisted subdivisions with sub-sampling to examine the impact of discrete sampling allocation and sample numbers per locations on our phylogeographic estimates. We note that sample sizes may strongly impact ancestral reconstruction using phylogenetic diffusion models. Depending on the location-specific diversity, over - and underrepresented locations may be more likely to be inferred as source/origin and

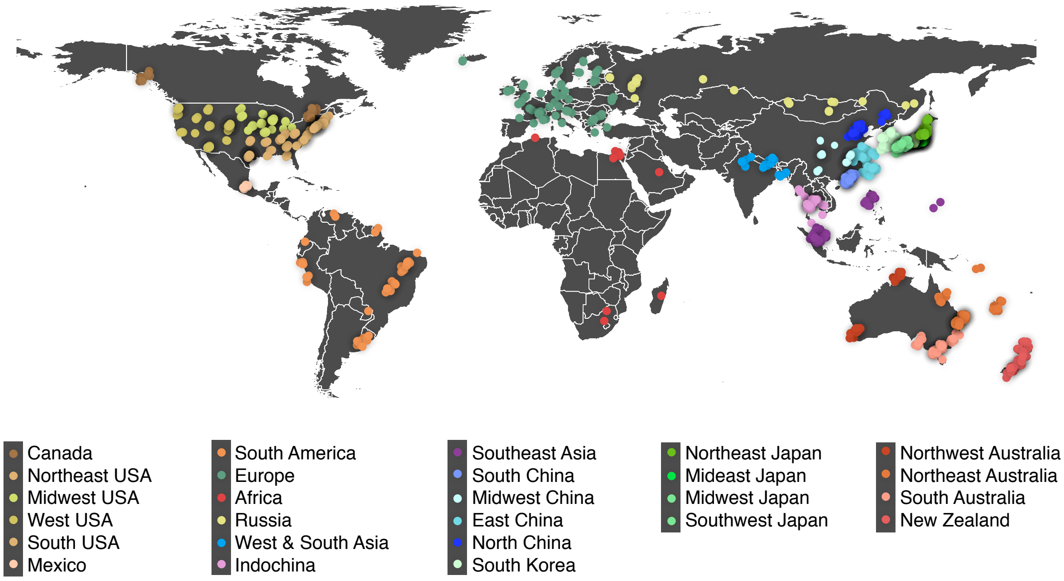

Figure S 1: **H3N2 sequence locations partitioned into 26 global geographic regions.** When the exact origin within a country was unknown, the sequence was mapped to the capital.

sink/destination locations respectively. The bias arising from such over - and underrepresentation may be more pronounced when the overall sampling is sparse because the location specific-diversity may be poorly captured in such cases. Although it may be useful to have more samples available from locations in which a large viral diversity has been established, which may for example help to establish its potential role as a source location, and this would be the case for a random sample from the entire population of infections, it remains difficult to assess to what extent convenient sampling reflects the underlying epidemiology. In an attempt to include all sequence data while keeping the number of samples per location as balanced as possible, we divided all the sequences into 26 geographic regions (Table S1 in Text S1) (Figure S1 in Text S1).

Since these spatial partitions sometimes required arbitrary subdivisions (e.g. breaking up USA, China, Japan and Australia), we also reduce this spatial partitioning to 15 geographic regions by joining regions from a single country (Table S2 in Text S1: Mideast Japan, Midwest Japan, Northeast Japan and Southwest Japan into Japan; East China, Midwest China, South China and North China into China; Northeast USA, West USA, South USA and Midwest USA into USA; and Northeast Australia, Northwest Australia and South Australia into Australia). Within each sampling year, we randomly down-sampled the five locations with the highest number of samples relative to their population size (USA: from 278 to 150; Australia: from 166 to 30; New Zealand: from 59 to 20; Japan: from 341 to 75; South Korea: from 51 to 30) and analyzed three different sub-sampled data sets. We note that trying to keep the number of samples per location as balanced as possible results in geographic partitions of various sizes, and more comprehensive sampling may enable more appropriate geographic partitioning in the future.

Because of the difficulties associated with geographic partitioning, we also identified

Table S 1: **Absolute latitude, (urban) population size, (urban) population density, H3N2 sequence sample sizes and antigenic residuals for 26 global geographic regions.** Antigenic residual estimates are described in section 1.3.2.

Region	Absolute latitude (degrees)	Population size	Population density (people per km^2)	Urban population size	Urban population density (people associated airport)	H3N2 sample size	Antigenic residual
Africa[1]	14.03	1.96E+08	26.67	6.56E+07	2.12E+06	10	-1.18
Canada[2]	50.67	3.23E+07	2.82	2.64E+07	8.50E+05	27	0.18
Europe	50.34	4.84E+08	96.18	2.80E+08	1.14E+06	80	-0.51
Indochina[3]	13.83	7.96E+07	114.72	1.22E+07	9.37E+05	59	-0.30
New Zealand	42.03	4.03E+06	14.89	3.24E+06	2.94E+05	59	0.07
Russia[4]	53.36	1.47E+08	7.86	5.31E+07	2.31E+06	24	-0.92
East China[5]	28.3	3.06E+08	453.29	5.83E+07	2.24E+06	56	0.57
Mexico	19.43	1.03E+08	52.49	5.60E+07	1.81E+06	10	0.19
Mideast Japan	35.71	4.54E+07	895.36	4.20E+07	2.10E+07	86	-0.03
Midwest China	29.82	3.08E+08	159.11	3.07E+07	2.36E+06	42	1.44
Midwest Japan	35.18	3.52E+07	573.15	2.75E+07	3.93E+06	80	-0.13
North China	40.27	1.24E+08	308.49	2.90E+07	4.84E+06	33	0.91
Northeast Australia	25.35	4.60E+06	2.42	4.06E+06	5.08E+05	79	-0.91
Northeast Japan	39.43	1.77E+07	108.6	1.36E+07	8.02E+05	82	-0.19
Northeast USA[6]	41.15	5.45E+07	129.59	3.74E+07	1.34E+06	48	0.64
Northwest Australia	24.72	2.15E+06	0.55	2.40E+06	1.20E+06	27	0.17
West USA[6]	40.34	3.20E+07	12.13	5.33E+07	1.18E+06	74	0.19
South America	15.73	3.12E+08	20.71	1.49E+08	1.71E+06	57	-0.57
West & South Asia[7]	27.17	1.28E+09	369.15	1.26E+08	4.06E+06	20	-2.33
South Australia	36.77	1.35E+07	6.48	1.29E+07	1.62E+06	60	-0.74
South China	22.33	1.02E+08	563.84	2.89E+07	3.21E+06	50	0.89
South Korea	36.17	4.73E+07	475.35	3.89E+07	3.89E+06	52	-0.08
Southeast Asia[8]	5.47	1.19E+08	169.04	5.43E+07	2.01E+06	58	0.19
South USA[6]	33.61	1.16E+08	51.59	5.48E+07	7.12E+05	104	0.50
Southwest Japan	34.02	2.51E+07	275.43	1.73E+07	9.59E+05	93	-0.36
Midwest USA[6]	42.02	5.30E+07	44.78	3.25E+07	7.23E+05	62	0.28

[1] includes Algeria, Egypt, Madagascar, South Africa and Saudi Arabia
[2] includes Canada and Alaska
[3] includes Cambodia and Thailand
[4] includes Russia and Mongolia
[5] includes Taiwan
[6] USA is partitioned according to the US census bureau regions
[7] includes India, Nepal and Bangladesh
[8] includes Philippines, Singapore, Malaysia and Guam

discrete air communities in the worldwide air transportation network and applied these as location states to our sequence sample. To increase sequence numbers for under-sampled air communities, we complemented the hemagglutinin gene sequences with publicly available sequences from Africa ($n = 21$), USA (Hawaii, $n = 4$), Central America ($n = 13$), South America ($n = 46$) and Canada ($n = 10$). From this data set, we removed six sequences that appeared to be outliers in a root-to-tip divergence versus sampling time regression analysis, resulting in a total of 1529 sequences. Within each sampling year, we randomly down-sampled the four locations with the highest number of samples relative to their population size (USA: from 318 to 120; Oceania: from 225 to 50; Japan: from 327 to 75; Southeast Asia: from 175 to 100; Table S3 in Text S1) and analyzed three different sub-sampled data sets discretized according to the 14 air communities. To asses the impact of sample sizes on the diffusion predictor identification (see below), we also perform the analysis on the complete data set on the one hand and the two randomly subsampled data sets on the other hand for which we restrict the number of sequences per location to 25 (Fig. S2). These scenarios

Table S 2: **Absolute latitude, population size, population density, H3N2 sequence sample and sub-sample sizes and antigenic residuals for 15 global geographic regions.**

Region	Absolute latitude (degrees)	Population size	Population density (people per km^2)	Urban population size	Urban population density (people per associated airport)	Agglomeration Index	H3N2 sample size	Antigenic residual
Africa	14.03	1.96E+08	26.67	65611208	2116491	59.2	10	-1.18
Canada	50.67	3.23E+07	2.82	26364443	850466	71	27	0.18
Europe	50.34	4.84E+08	96.18	279955859	1142677	62.5	80	-0.51
Indochina	13.83	7.96E+07	114.72	12180317	936947	30.85	59	-0.30
New Zealand	42.03	4.03E+06	14.89	3235714	294156	66	59/20[1]	0.07
Russia	53.36	1.47E+08	7.86	53130040	2310002	63	24	-0.92
China	29.19	8.40E+08	263.06	146832329	2719117	36.2	181	0.92
Japan	36.01	1.23E+08	336.98	100388251	2281551	92.9	341/75[1]	-0.18
Australia	29.38	2.02E+07	2.57	19400265	1077793	75.2	166/30[1]	-0.70
USA	38.35	2.56E+08	39.37	178056518	913110	72.3	278/150[1]	0.39
Mexico	19.43	1.03E+08	52.49	55986623	1806020	66.7	10	0.19
South America	15.73	3.12E+08	20.71	149034939	1713045	60.37	58	-0.57
South Asia	27.17	1.28E+09	369.15	125459433	4181981	39.7	20	-2.33
South Korea	36.17	4.73E+07	475.35	38913352	3891335	89.6	51/30[1]	-0.08
Southeast Asia	5.47	1.19E+08	169.04	54333014	2012334	75.03	58	0.19

[1] Sample sizes are provided before/after down-sampling

represent more imbalanced and more balanced sample sizes respectively with respect to the three sub-sampled data sets used throughout the main text.

Table S 3: **Absolute latitude, (urban) population size, (urban) population density, agglomeration index, H3N2 sequence sample and sub-sample sizes and antigenic residuals for 14 global air communities.**

Region	Absolute latitude (degrees)	Population size	Population density (people per km^2)	Urban population size	Urban population density (people per associated airport)	Agglomeration Index	H3N2 sample size	Antigenic residual
Africa	22.27	8.03E+07	44.53	2.53E+07	2.53E+06	38.3	23	-0.93
USA	37.33	2.95E+08	32.21	2.51E+08	9.39E+05	72.3	318/120[1]	0.34
Taiwan[2]	25.04	2.28E+07	629.54	1.32E+07	1.46E+06	84.4	17	0.25
China	32.42	1.29E+09	134.88	1.62E+08	2.61E+06	36.2	122	0.88
Russia	55.6	1.44E+08	8.44	8.68E+07	1.61E+06	63	17	-1.05
Oceania	32.69	2.46E+07	3.06	2.27E+07	7.10E+05	49.83	225/50[1]	-0.51
West & South Asia	27.61	1.38E+09	208.34	1.88E+08	3.24E+06	58.2	26	-0.11
Japan	36.03	1.28E+08	342.62	9.57E+07	2.08E+06	92.9	327/75[1]	-0.22
Mexico	19.65	1.03E+08	52.49	5.84E+07	1.83E+06	66.7	12	0.46
South America	18.02	3.12E+08	20.71	1.35E+08	1.73E+06	62.9	101	-0.32
Canada	48.53	3.16E+07	3.17	2.60E+07	9.30E+05	71	24	0.11
Europe	48.44	4.84E+08	96.18	2.64E+08	1.11E+06	63.4	85	-0.58
Southeast Asia	15.27	2.06E+08	69.71	8.11E+07	1.62E+06	65.13	175/100[1]	0.10
South Korea	36.01	4.73E+07	475.35	3.92E+07	2.53E+06	89.6	46	-0.04

[1] Sample sizes are provided before/after down-sampling
[2] In the 26-region and 15-region geographic partitioning, the 17 Taiwanese sequences were included with East China and China respectively.

1.2 Incorporating uncertainty in air community assignment

Using a generalization of the method introduced in [2], we identified highly modular partitions in the global air transportation network. For an ensemble of 1000 modularity subdivisions we quantify the uncertainty by an affinity matrix that, for each pair of locations, summarizes the fraction of partitions in which these locations are in the same community. Based on a partition encompassing a number of air communities ($n = 14$) that is in size close

to the 15-geographic region partition, we subsequently obtain the average affinity for each airport to the communities in this partition. We assign each airport to the community for which it shows the highest average affinity, but we take into account its uncertainty by also considering assignments that yield affinities that are > 2/3 of the highest affinity score. This cut-off resulted in 771 ambiguous airport assignments. Finally, we partitioned the sequence data according to the air community assignment and accommodate 368 (24%) ambiguous sequence locations, i.e. those sequences related to airports with ambiguous community assignments, using ambiguity coding in our phylogeographic approach.

1.3 Bayesian statistical analysis of sequence and trait evolution

We integrate genetic, spatial and air transportation data within a single full probabilistic evolutionary model and simultaneously estimate the parameters of phylogeographic diffusion using Markov chain Monte Carlo (MCMC) analysis implemented in BEAST [3]. We introduce a novel phylogenetic diffusion model and associated inference procedures in the subsections below. To model sequence evolution, we partition the hemagglutinin codon positions into first+second and third positions [4] and apply a separate HKY85 [5] CTMC model of nucleotide substitution with discrete gamma-distributed rate variation [6] to both. We assume a flexible Bayesian skyride prior over the unknown phylogeny [7]. Exploratory runs using the data for the 26 locations indicated that a relaxed molecular clock represented an over-parametrization [8]. A strict clock was therefore used in subsequent analyses. Because the exact date of sampling was not known for some additional publicly available sequences, we integrated out their dates over the known sampling time interval [9]. We capitalize on BEAGLE [10] in conjunction with BEAST to improve computational performance on our large data sets. MCMC analyses were run sufficiently long to ensure stationarity as diagnosed using Tracer. We used the TreeAnnotator tool in BEAST to summarize trees in the form of maximum clade credibility (MCC) trees. As part of the supporting information files (Dataset S1), we make available an XML document specifying the data and analysis settings for main analysis of the air communities, and the associated empirical trees required to run the analysis (section 1.3.3). This includes accession numbers for all the sequences as well as their sampling dates, the locations we assigned them to (section 1.1), the different sub-samplings, the (GLM) model settings and the predictors (section 1.3.1 and 1.3.2).

1.3.1 GLM diffusion implementation and predictor support

Bayesian phylogeographic inference models discrete diffusion as a continuous-time Markov chain process parameterized in terms of a $K \times K$ infinitesimal rate matrix $\mathbf{\Lambda}$ of discrete location change with K representing the number of location states. The GLM diffusion model extends this by adopting a generalized linear model (GLM) approach that takes an arbitrary number P of predictors $\mathbf{X} = (\mathbf{x}_1, \ldots, \mathbf{x}_P)$, where a single predictor $\mathbf{x}_p$ is a flattened vector of quantities corresponding to entries in the i to j rate matrix $\mathbf{x}_p = (x_{1,2,p}, \ldots x_{K-1,K,p})'$. The GLM considers every instantaneous movement rate Λ_{ij} for $i \neq j$ in $\mathbf{\Lambda}$ as a log linear function of the set of predictors $\mathbf{X}$, such that:

$$\log \Lambda_{ij} = \beta_1 \delta_1 x_{i,j,1} + \beta_2 \delta_2 x_{i,j,2} + \ldots + \beta_P \delta_P x_{i,j,P}, \tag{1}$$

where $\boldsymbol{\beta} = (\beta_1, \ldots, \beta_P)'$ represent the effective sizes for the predictors, quantifying their contribution to $\boldsymbol{\Lambda}$, and $(\delta_1, \ldots, \delta_P)$ are (0,1)-indicator variables that govern the inclusion or exclusion of the P predictors in the model. In constructing our predictors, we shift the covariates to ensure positivity if needed (e.g. using a pseudo count for the passenger flux) and subsequently log-transform them. We note that other transformations, e.g. from the Box-Cox family, may also be explored in our framework. The incorporation of indicator variables allows for Bayesian stochastic search variable selection (BSSVS) [11, 12], which involves estimating the posterior probabilities of all 2^P possible linear models that may or may not include the predictors. When an indicator δ_p equals 1, then predictor $\mathbf{x}_p$ is included in the model, demonstrating that it helps to explain the diffusion process in the phylogenetic history with high probability. We complete this GLM specification with variable selection by assigning independent Bernoulli prior probability distributions on δ_p. We use a small prior probability on each predictor's inclusion that reflects a 50% prior probability on no predictors being included, but specifying equal prior probability on each predictor's inclusion and exclusion yields highly similar results (Fig. S3). Lemey et al. [13] discuss BSSVS in further detail and analogous to Edo-matas et al. [14], we can use Bayes factors (BFs) [15, 16] to express how much the data change our prior opinion about the inclusion of each predictor. These BFs are calculated by dividing the posterior odds for the inclusion of a predictor with the corresponding prior odds, e.g. 0.019:0.981 prior odds for the analysis of the 14 air communities and 15 geographic regions (Fig. 2 in the main manuscript), or 1:1 odds for the same analyses using equal prior probability for each predictor's inclusion and exclusion (Fig. S3):

$$\mathrm{BF}_p = \frac{\mathrm{pp}_p}{1 - \mathrm{pp}_p} / \frac{\mathrm{qp}_p}{1 - \mathrm{qp}_p}, \tag{2}$$

where pp_p is the posterior probability that predictor p is included, in this case the posterior expectation of indicator δ_p, and qp_p is the prior probability that $\delta_p = 1$. The posterior odds follows immediately from the marginal posterior probability that a predictor indicator equals 1, estimated through the posterior expectation of the predictor indicator. We specify that *a priori* all β_p are independent and normally distributed with mean 0 and a relatively large variance of 4, which still ensures adequate mixing. We implement the GLM-diffusion parametrization in the software package BEAST [3] and approximate the joint posterior and its marginalizations using standard Markov chain Monte Carlo (MCMC) transition kernels. Similar to recent advances in phylogeographic inference in continuous space [17], we integrate out discrete location states at internal nodes in the trees but draw stochastic realizations of the node states when logging the trees. An important, novel extension to the standard MCMC machinery in BEAST lies in generating an efficient Metropolis-Hastings proposal distribution for the GLM coefficients $\boldsymbol{\beta}$. Given the potential for high correlation between predictors $\mathbf{X}$, attempting to update one coefficient β_p at a time while holding the remaining coefficients constant returns high autocorrelations times. Instead, we exploit the fixed correlation structure $\mathbf{X}'\mathbf{X}$ between predictors to generate a multivariate proposal $\boldsymbol{\beta}^\star$. In particular, if we assume $\boldsymbol{\beta}$ are the current realized values, then we draw

$$\boldsymbol{\beta}^\star \sim \text{Multivariate-Normal}\left(\boldsymbol{\beta}, \alpha \left(\mathbf{X}'\mathbf{X}\right)^{-1}\right), \tag{3}$$

where α is an auto-tunable variance scalar. Motivation for this proposal stems from imagining that the marginal posterior distribution of $\boldsymbol{\beta}$ under our phylogenetic GLM should partially approximate a simple linear regression model involving $\boldsymbol{\beta}$, whose posterior variance is proportional to $\mathbf{X}'\mathbf{X}$. We note that colinearity among explanatory variables is not allowed because this would make the $\mathbf{X}'\mathbf{X}$ singular and not invertible. We capture the numerical exception that our transition kernel for the regression coefficients would throw in this case, which will inform the user about colinearity issues. We consider a 'bit-flip' operator on the Bernoulli rate indicators; this transition kernel is further discussed in [18].

1.3.2 GLM diffusion predictors

Depending on the location state partitioning scheme, we considered several potential predictors of global influenza diffusion in the GLM diffusion model:

- **Average and minimum distance**. To test whether geographical proximity predicts influenza diffusion we considered two different great-circle distance measures: (i) the average distance between two locations based on the pairwise distances between all pairs of airports from the two locations and (ii) the minimum distance amongst those pairwise distances.

- **Absolute latitude**. Absolute latitudes for each region/community were calculated as the absolute values of the average latitudes of the sequence sampling locations (Sequences from unknown locations within specific countries were assigned to the capital of that country) and are listed in Table S1, S2 and S3 in Text S1.

- **Passenger flux**. The total number of seats on flights between each pair of locations per day. In addition, we also include a separate origin and destination predictor that summarizes the total air flux within each air community or geographic regions, thus representing the within-location air connectivity. Although the passenger flux data does not account for variations in passenger occupancy, the seat-based predictors are expected to be fairly robust to this because the variation in actual fluxes across the links in the air transportation network are orders of magnitude higher than variations between occupancy and seat numbers. Because passenger flux does not differ in a statistically significant manner from symmetry in the global air transportation network [19], we consider flows that were symmetrized.

- **Population size and density**. Population size estimates for 2005, or interpolated for 2005 based on data close to this year, were obtained from Geographica [20] or `www.citypopulation.de` (listed in Table S1, S2 and S3 in Text S1). In addition to general population sizes/densities, we also consider urban population sizes for the air communities/geographic regions based on airport-associated cities. For this purpose, we downloaded population sizes for all cities with a population exceeding 15000 (downloaded from the GeoNames geographical database: www.geonames.org) and associated them with the closest airport from the 1227-largest-airport network (that represents 95% of the passenger flux) within a 75 km radius. We subsequently aggregated all these airport associated population sizes for each air community or geographic area in

which the airports are located and also included these as potential predictors in the model. This measure does not appear to be very sensitive to the radius threshold we use to associate cities with airports because a radius of 200 km results in numbers that highly correlate with the urban population sizes we used ($r > 0.97$). As an urban population density measure that accounts for the number of airports these population sizes are distributed over, we also considered a predictor that divides the airport-associated urban population sizes by the number of airports to which they were assigned in each air community/geographic region. Both the urban population sizes and densities are also listed in Table S1, S2 and S3 in Text S1. We report the GLM estimates for the general population sizes and densities (Fig. 2 in the main manuscript and Fig. S3), but list the estimates for the full set of predictors for each sub-sampled data set in Fig. S1. In addition, we report estimates for the full set of predictors for the complete data set and for two random sub-samples that include a maximum of 25 sequences per location for the air communities (Fig. S2).

To include a measure of population clustering, we also incorporated an 'agglomeration Index (AI)', which is based on population density, population of large urban centers (>50,000 people) and travel time to that urban center [21]. This was included for the analysis of the 14-air community and 15-location geographic partitions (Table S2 and S3 in Text S1), but the level of detail was not available for the 26-location geographic partitions. For air communities or geographic regions for which sequences were available for a limited number of countries, we summarized average (urban) population sizes, densities and AIs based on only these countries. All demographic measures were included as separate origin and destination predictors.

- **Economic data – Gross domestic product (GDP).** We construct predictors based on GDP data collected for the year 2004 from the World Bank (data.worldbank.org/) If data was not available from this resource, we resorted to GeoHive (http://www.geohive.com) to complement it. Analogous to the demographic measures, we obtain GDP averages from countries for which sequence samples were available in case we only had samples available for only a limited number of countries per air community or geographic region. Analogous to the AIs, the GDP was available for the 14-air community and 15-location geographic partitions, but not to the level of detail required for the 26-location geographic partitions.

- **Viral surveillance data**. To test the predictive power of viral surveillance data, we essentially aimed at capturing the nature and degree of synchronicity of yearly incidence profiles in each air community/geographic region. To this purpose, we extracted the number of influenza viruses A(H3) detected per country from week 1 in 1997 to week 45 in 2010 from FluNet/WHO (`www.who.int/flunet`) for relevant countries in the 14-air community and 15-region geographic partition schemes. The level of detail retired for the 26-region geographic partition scheme was not available. Taiwanese surveillance data was obtained from [22]. We focused on the influenza A(H3) incidence counts between 2002 and 2007 or as close as possible to this time period when insufficient data was available. Average incidence counts were used when data from multiple countries per region/community was available. We subsequently calculated average incidences

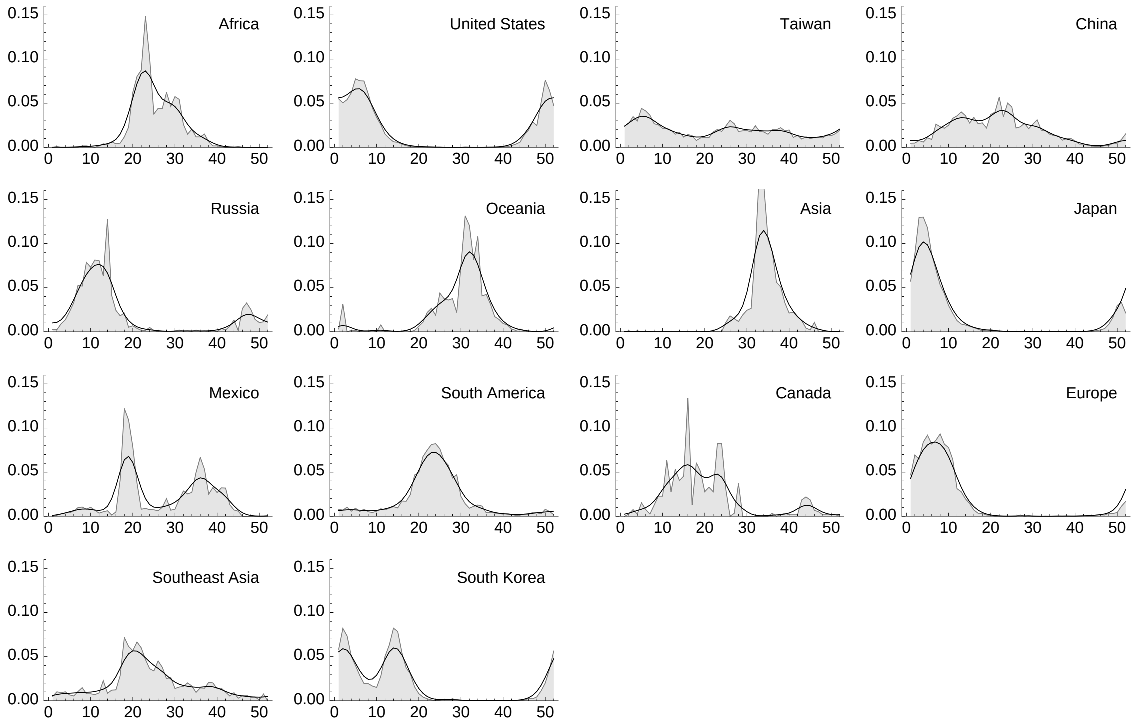

Figure S 2: **H3 incidence profiles for the 14 air communities based on both raw and smoothed, normalized, weekly average counts for relevant countries in the air communities.** For the following regions we include data from specific countries: Africa (South Africa), Oceania (Australia), South & West Asia (Bangladesh and India), South America (Argentina, Brazil, Chile, Ecuador, Paraguay, French Guiana, Peru, Uruguay and Venezuela), Europe (Bulgaria, Croatia, Czech Republic, Denmark, Finland, France, Germany, Greece, Iceland, Ireland, Italy, Latvia, Netherlands, Norway, Romania, Slovenia, Spain, Sweden, Switzerland, Turkey, Ukraine, UK and Ireland,) and Southeast Asia (Malaysia, Philippines, Singapore and Thailand).

per week across multiple years for each region/community, normalized these weekly averages and smoothed them with a Gaussian standard deviation of 2 weeks. Fig. S2 in Text S1 depicts the resulting incidence profiles for the 14 air communities.

We derived several potential predictors from these incidence profiles, including incidence overlap, origin incidence versus destination growth rate, peak time difference, and incidence in the origin location at fixed times prior to peak incidence in the destination location. The incidence overlap summarizes the overlapping area under the origin-destination incidence curves for each pair of locations. The origin incidence versus destination growth rate sums the product of origin incidence and destination growth rate for each week of the year. Peak time difference quantifies the difference in peak incidence for each origin-destination pair. For the latter, we summarized the donor incidences at 4, 8, 12, 16, 20 and 24 weeks prior to peak incidence in the destination location as potential predictors. We report the GLM estimates for the incidence overlap (Fig. 2 in the main manuscript and Fig. S3), which serves as a representative predictor for all incidence-derived measures. The estimates for the full set of predictors are summarized for each of the three sub-sampled data set in Fig. S1 and for the

complete data set and two smaller but more balanced sub-samples in Fig. S2.

In addition to including measures of synchronicity in epidemic profiles between pairs of locations, we also attempted to capture the seasonality of each air of the 14 air communities and 15 geographic partitions. In particular, we consider the origin and destination seasonal entropy, which is the degree of entropy in weekly incidence across the year for the origin/destination region. In this case, flatter distributions (like China, Taiwan and Southeast Asia) have larger entropies. As an alternative, we consider a predictor that quantifies the number weeks out of the year that have standardized incidence greater than the median incidence ('origin and destination above-median incidence').

- **Antigenic evolution**. Because antigenic evolution can provide insights into the seeding dynamics of seasonal H3N2 [1], we sought to include the average antigenic divergence for each region as phylogeographic diffusion predictors. Based on the available antigenic cartography data for the strains in our phylogeographic analyses, we performed a local regression (LOESS) of the principle antigenic component, obtained from a multidimensional scaling analysis of hemagglutination inhibition assay measurements [1], against time. The resulting scatter plot with strains colored according to air community is presented in Fig. S3 in Text S1. Distances from the spline (residuals) were calculated for each antigenic measurement and average residuals were obtained for each region/community, which reflects whether a location is on average antigenically leading or trailing [1]. These average residuals are listed in Table S1, S2 and S3 in Text S1. We considered the exponentiated residual and exponentiated negative residual as a measure of efflux and influx respectively for each location and included these as separate origin and destination predictors.

- **Sample sizes**. To test the impact of sampling effects, we considered origin and destination sample sizes (number of H3N2 sequences included per discrete location state in the phylogeographic analysis) as separate predictors. Although sampling sizes are expected to have an impact on the number of location transitions, support for other factors in addition to sampling size predictors may suggest that they are robust to potential sampling biases.

In constructing predictors, we log-transform all strictly positive quantities and then standardized all predictors to have a mean of 0 and a variance of 1 before their incorporation in the GLM approach. Standardization facilitates prior specification and BSSVS.

1.3.3 Fitting diffusion models to empirical tree distributions

Although we generally desire to simultaneously reconstruct sequence and discrete/continuous trait evolution using our Bayesian statistical framework, integrating over tree-space becomes a computationally daunting task for a large number of taxa. The main limiting factor in Bayesian analysis of evolutionary history is typically the efficiency with which topology proposals explore phylogenetic tree space [23]. To side-step these limitations, we seek to approximate phylogenetic uncertainty in our phylogeographic estimates in cases where sampling from tree space needs to be performed repeatedly (e.g. when comparing different

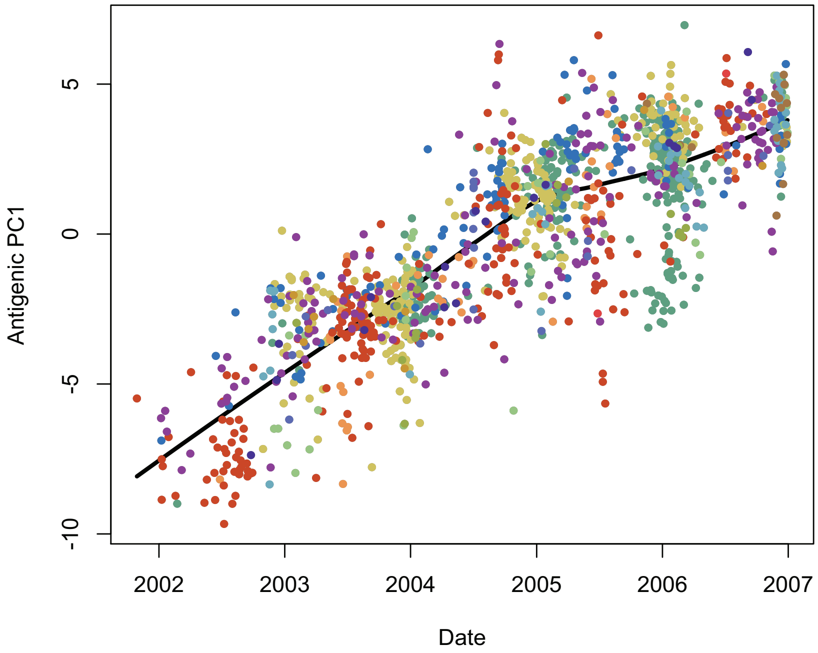

Figure S 3: **Scatter plot with local regression (LOESS) fit for the first principle component (PC1) of the H3N2 antigenic measurements against time.** Antigenic PC1 data points are colored according to the air communities represented in Fig. 1 in the main manuscript.

diffusion models). To this purpose, we follow [24] and implement a transition kernel that randomly draws from an empirical posterior distribution of trees which, in our case, were solely inferred from sequence data. Because the likelihood of a tree topology will largely be dominated by an informative sequence alignment compared to a single discrete (location) site, we expect such an empirical approach will closely approximate the phylogenetic uncertainty in the joint inference approach. We provide an example empirical tree set for one of the subsampled air community data sets in Dataset S1.

1.4 Comparing simulated spatial expansion to recorded H1N1 pandemic data

Based on the numbers of pandemic H1N1 isolates detected per week per country during 2009, downloaded from the World Health Organization database FluNet (www.who.int/flunet), we combine time series between countries to estimate regional patterns. To control for sampling intensity, detection counts are adjusted so that the total number of isolates is equal between countries. The resulting counts are further adjusted by scaling in proportion to the population size of each country. The estimates for each region are only marginally affected by these adjustments (moving the initial epidemic peak by at most one week). To control for week-to-week stochastic variation in sampling intensity, we smooth each regional distribution using kernel density estimation with a Gaussian kernel and a 10-day bandwidth. The resulting time series often show multiple distinct peaks, with many temperate regions showing a smaller summer peak and a later more-severe fall peak (Fig. S4 in Text S1). We

are primarily interested in the initial spread of the pandemic and so take the timing of the initial peak as an indication of the rate and pattern of geographic spread.

We run simulations for different migration matrices and compare simulated epidemic peaks to observed initial peaks in the FluNet data (Fig. 4 in the main text for simulations using an equal rate matrix, air travel data, standard phylogeographic estimates and GLM estimates respectively, and Fig. S5 for simulations using a migration matrix estimated by BSSVS). We calculate the peak incidence of each regional epidemic across 100 simulations, yielding a mean and 95% range for the timing of each peak in each region, and measure the relative correspondence between the mean peak times and the observed peak times for all locations except Mexico using the Spearman's rank correlation coefficient. The significance test takes the mean peak times across the simulations and the mean observed peak times, and then randomly permutates their values across the regions to assess in what frequency we get more extreme Spearman rank correlations than the original correlation value.

2 Evaluation of the GLM-difussion approach on empirical data

As a validation, we test the GLM extension of the discrete diffusion model by simultaneously inferring the phylogeographic history of dog rabies viruses in Morocco and identifying the factors driving their dispersal based on a previously published data set [25]. The original study evaluated several potential predictors of dog rabies dispersal by fixing the parameters of the discrete rate matrix and independently fitting different models to the joint sequence and spatial data. Model-fit, as assessed using a harmonic mean estimator (HME) of the marginal likelihood, indicated that road distances were the best predictors of rabies diffusion in Morocco [25]. Considering the large variance and poor repeatability of the HME (see e.g. [26]) and most importantly the fact that the HME systematically overestimates the marginal likelihood [27], it remains difficult to unambiguously reject competing hypotheses (such as geographic distances, which were less than one ln likelihood unit different from the best fitting model).

Table S 4: **Model-fit for different predictors of dog rabies diffusion in Morocco**. The best-fitting model according to each model comparison approach is indicated in bold.

	Fitting (1)								Fitting (2)							
Predictor	HME	rank	AICM	rank	PS	rank	SSS	rank	HME	rank	AICM	rank	PS	rank	SSS	rank
Equal rates	-10220.8	(4)	20714.4	(4)	-10823.3	(4)	-10824.6	(4)	-10220.2	(4)	20715.8	(4)	-10822.5	(4)	-10823.7	(4)
Great circle distances	**-10199.8**	**(1)**	**20664.3**	**(1)**	-10810.5	(2)	-10811.9	(2)	-10201.4	(2)	20684.9	(2)	-10803.9	(2)	-10805.2	(2)
Population sizes	-10281.8	(6)	20859.9	(6)	-10886.6	(6)	-10888.0	(6)	-10281.3	(6)	20845.1	(6)	-10882.4	(6)	-10883.8	(6)
Gravity model	-10287.9	(7)	20865.2	(7)	-10897.7	(7)	-10899.0	(7)	-10287.9	(7)	20865.7	(7)	-10895.3	(7)	-10896.6	(7)
Population surface	-10235.9	(5)	20746.2	(5)	-10842.7	(5)	-10844.1	(5)	-10238.9	(5)	20749.5	(5)	-10827.6	(5)	-10829.2	(5)
Road distances	-10200.4	(2)	20677.2	(2)	**-10809.2**	**(1)**	**-10810.5**	**(1)**	**-10199.3**	**(1)**	**20670.3**	**(1)**	**-10799.1**	**(1)**	**-10800.4**	**(1)**
Accessibility	-10214.3	(3)	20701.8	(3)	-10822.2	(3)	-10823.5	(3)	-10215.4	(3)	20703.1	(3)	-10816.0	(3)	-10817.3	(3)

Here we compare our GLM-diffusion model with different marginal likelihood estimators including the HME and recent BEAST implementations of path sampling (PS), stepping stone sampling (SS) [28, 29, 30, 27, 31], as well as the Akaike's information criterion through MCMC (AICM,[28, 32]). Table S4 lists the marginal likelihood estimates for rates fixed to equal values, inverse distances, the product of origin and destination population sizes

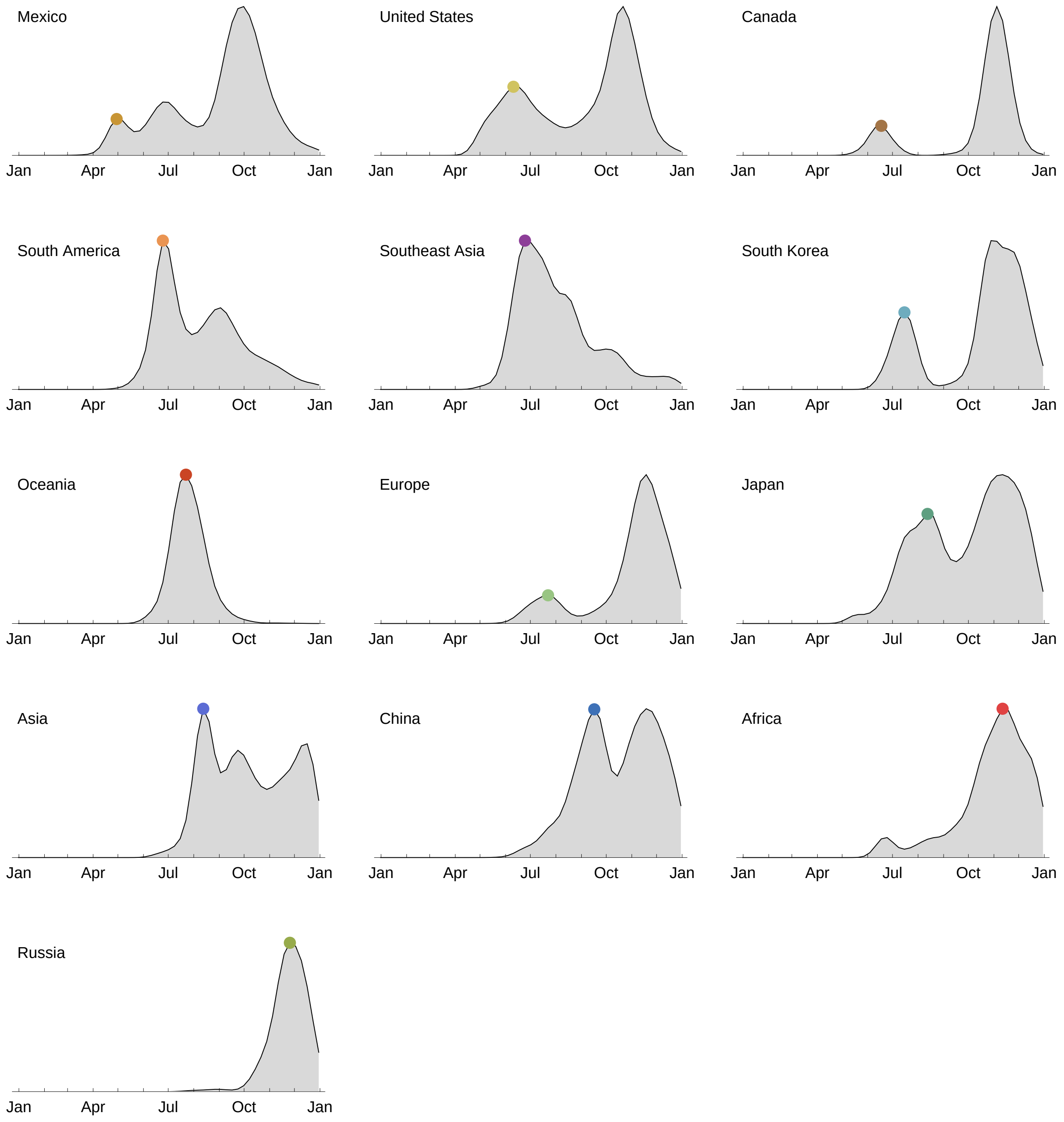

Figure S 4: **Regional time series of pandemic H1N1 isolations during 2009.** Shown are the entire distributions of isolations as black lines and the initial peaks as colored points. Regions are ordered based on the occurrence of these initial peaks.

Table S 5: **Predictors of dog rabies diffusion in Morocco.** Bayes factors formalize the support for the predictor whereas the conditional effect sizes (on log scale) reflect the contribution of the predictor when they are included in the model.

Predictor	Bayes Factor	Conditional Effect size
Distance	0.51	-0.15 (-1.58, 2.01)
Donor population size	0.07	-0.09 (-0.37, 0.16)
Recipient population size	0.05	0.08 (-0.16, 0.25)
Population surface	0.45	-0.45 (-0.94, -0.04)
Road distances	6.64	-1.37 (-2.86, -0.69)
Accessibility	0.09	-0.16 (-0.57, 0.21)

(human population sizes as a proxy for dog population sizes), the product of origin and destination population sizes divided by geographic distance (the so-called gravity model), inverse population surface distances, inverse road distances and accessibility estimates [25]. Two independent analyses were performed to assess the variability of the different estimators. The two runs for PS and SS consistently favored road distance over the other predictors while the HME and AICM preferred either road or geographic distance. Formal evaluation of these model comparison approaches has recently shown that both PS and SS are more reliable and more consistent than the HME and the AICM [28, 29, 30]. Nevertheless, substituting marginal likelihood estimates between the two runs for some predictors could yield a different PS or SS ranking of the models indicating that unambiguous model selection remains difficult.

When applying the GLM-diffusion model, we do not consider equal rates because the model can default to this scenario when no predictors are included. We also do not explicitly construct a product of origin and population sizes nor a gravity model because the GLM approach can invoke these by simultaneously including the individual predictors (origin population size, destination population size and distance). The GLM-diffusion results in Table S 5 indicate that only road distances are supported (BF= 6.64) as a predictor with a mean conditional effect size of about -1.37 on a log scale and credible intervals that do not include zero. In conclusion, the GLM-diffusion model is consistent with state-of-the-art marginal likelihood estimators and direct support for predictors allows more robust model selection when comparing similar models.

3 References

[1] Russell CA, Jones TC, Barr IG, Cox NJ, Garten RJ, et al. (2008) The global circulation of seasonal influenza a (H3N2) viruses. Science 320: 340–346.

[2] Guimerà R, Mossa S, Turtschi A, Amaral LAN (2005) The worldwide air transportation network: Anomalous centrality, community structure, and cities' global roles. Proc Natl Acad Sci U S A 102: 7794-9.

[3] Drummond AJ, Suchard MA, Xie D, Rambaut A (2012) Bayesian phylogenetics with BEAUti and the BEAST 1.7. Mol Biol Evol 29: 1969-73.

[4] Shapiro B, Rambaut A, Drummond AJ (2006) Choosing appropriate substitution models for the phylogenetic analysis of protein-coding sequences. Mol Biol Evol 23: 7-9.

[5] Hasegawa M, Kishino H, Yano T (1985) Dating the human-ape splitting by a molecular clock of mitochondrial DNA. Journal of Molecular Evolution 22: 160–174.

[6] Yang Z (1995) A space-time process model for the evolution of DNA sequences. Genetics 139: 993–1005.

[7] Minin V, Bloomquist E, Suchard M (2008) Smooth skyride through a rough skyline: Bayesian coalescent-based inference of population dynamics. Mol Biol Evol 25: 1459-71.

[8] Drummond A, Ho S, Phillips M, Rambaut A (2006) Relaxed phylogenetics and dating with confidence. PLoS Biology 4: e88.

[9] Shapiro B, Ho SYW, Drummond AJ, Suchard MA, Pybus OG, et al. (2011) A bayesian phylogenetic method to estimate unknown sequence ages. Mol Biol Evol 28: 879-87.

[10] Suchard MA, Rambaut A (2009) Many-core algorithms for statistical phylogenetics. Bioinformatics 25: 1370-6.

[11] Kuo L, Mallick B (1998) Variable selection for regression models. Sankhya B 60: 65–81.

[12] Chipman H, George E, McCulloch R (2001) The practical implementation of Bayesian model selection. IMS Lecture Notes – Monograph Series 38: 67–134.

[13] Lemey P, Rambaut A, Drummond A, Suchard M (2009) Bayesian phylogeography finds its root. PLoS Computational Biology 5: e1000520.

[14] Edo-Matas D, Lemey P, Tom JA, Serna-Bolea C, van den Blink AE, et al. (2011) Impact of CCR5delta32 host genetic background and disease progression on HIV-1 intrahost evolutionary processes: efficient hypothesis testing through hierarchical phylogenetic models. Mol Biol Evol 28: 1605-16.

[15] Jeffreys H (1998) Theory of Probability. Oxford Classic Texts in the Physical Sciences. New York: Oxford University Press, 3rd edition.

[16] Suchard M, Weiss R, Sinsheimer J (2001) Bayesian selection of continuous-time Markov chain evolutionary models. Molecular Biology and Evolution 18: 1001–1013.

[17] Pybus OG, Suchard MA, Lemey P, Bernardin FJ, Rambaut A, et al. (2012) Unifying the spatial epidemiology and molecular evolution of emerging epidemics. Proc Natl Acad Sci U S A 109: 15066-71.

[18] Drummond AJ, Suchard MA (2010) Bayesian random local clocks, or one rate to rule them all. BMC Biol 8: 114.

[19] Woolley-Meza O, Thiemann C, Grady D, Lee J, Seebens H, et al. (2011) Complexity in human transportation networks: a comparative analysis of worldwide air transportation and global cargo-ship movements. Eur Phys J B 84: 589-600.

[20] Sandall P (2006) Geographica: the complete illustrated atlas of the world. Jill Baker, Random House Australia Pty. Ltd.

[21] Uchida H, Nelson A (2008) Agglomeration index: Towards a new measure of urban concentration. Technical report, Washington : World Bank.

[22] Lin JH, Chiu SC, Cheng JC, Chang HW, Hsiao KL, et al. (2011) Molecular epidemiology and antigenic analyses of influenza a viruses h3n2 in taiwan. Clin Microbiol Infect 17: 214-22.

[23] Lakner C, van der Mark P, Huelsenbeck JP, Larget B, Ronquist F (2008) Efficiency of markov chain monte carlo tree proposals in bayesian phylogenetics. Syst Biol 57: 86-103.

[24] Pagel M, Meade A, Barker D (2004) Bayesian estimation of ancestral character states on phylogenies. Systematic Biology 53: 673–684.

[25] Talbi C, Lemey P, Suchard MA, Abdelatif E, Elharrak M, et al. (2010) Phylodynamics and human-mediated dispersal of a zoonotic virus. PLoS Pathog 6: e1001166.

[26] Fan Y, Wu R, Chen M, Kuo L, Lewis P (2011) Choosing among partition models in Bayesian phylogenetics. Molecular Biology and Evolution 28: 523–532.

[27] Lartillot N, Philippe H (2006) Computing Bayes factors using thermodynamic integration. Systematic Biology 55: 195–207.

[28] Baele G, Lemey P, Bedford T, Rambaut A, Suchard M, et al. (2012) Improving the accuracy of demographic and molecular clock model comparison while accommodating phylogenetic uncertainty. Molecular Biology and Evolution 29: 2157-2167.

[29] Baele G, Li WLS, Drummond AJ, Suchard MA, Lemey P (2013) Accurate model selection of relaxed molecular clocks in bayesian phylogenetics. Molecular Biology and Evolution 30: 239-243.

[30] Baele G, , Lemey P (2013) Bayesian evolutionary model testing in the phylogenomics era: matching model complexity with computational efficiency. Bioinformatics 29: 1970-1979.

[31] Xie W, Lewis P, Fan Y, Kuo L, Chen M (2011) Improving marginal likelihood estimation for Bayesian phylogenetic model selection. Systematic Biology 60: 150–160.

[32] Raftery A, Newton M, Satagopan J, Krivitsky P (2007) Estimating the integrated likelihood via posterior simulation using the harmonic mean identity. In: JMBernardo, Bayarri M, Berger J, editors, Bayesian Statistics, Oxford University Press. pp. 1–45.
